# The role of loop dynamics in the prediction of ligand–protein binding enthalpy†

**DOI:** 10.1039/d2sc06471e

**Published:** 2023-06-01

**Authors:** Süleyman Selim Çınaroğlu, Philip C. Biggin

**Affiliations:** a Structural Bioinformatics and Computational Biochemistry, Department of Biochemistry, University of Oxford South Parks Road Oxford OX1 3QU UK philip.biggin@bioch.ox.ac.uk +44 (0)1865 613238 +44 (0)1865 613305

## Abstract

The enthalpic and entropic components of ligand–protein binding free energy reflect the interactions and dynamics between ligand and protein. Despite decades of study, our understanding and hence our ability to predict these individual components remains poor. In recent years, there has been substantial effort and success in the prediction of relative and absolute binding free energies, but the prediction of the enthalpic (and entropic) contributions in biomolecular systems remains challenging. Indeed, it is not even clear what kind of performance in terms of accuracy could currently be obtained for such systems. It is, however, relatively straight-forward to compute the enthalpy of binding. We thus evaluated the performance of absolute enthalpy of binding calculations using molecular dynamics simulation for ten inhibitors against a member of the bromodomain family, BRD4-1, against isothermal titration calorimetry data. Initial calculations, with the AMBER force-field showed good agreement with experiment (*R*^2^ = 0.60) and surprisingly good accuracy with an average of root-mean-square error (RMSE) = 2.49 kcal mol^−1^. Of the ten predictions, three were obvious outliers that were all over-predicted compared to experiment. Analysis of various simulation factors, including parameterization, buffer concentration and conformational dynamics, revealed that the behaviour of a loop (the ZA loop on the periphery of the binding site) strongly dictates the enthalpic prediction. Consistent with previous observations, the loop exists in two distinct conformational states and by considering one or the other or both states, the prediction for the three outliers can be improved dramatically to the point where the *R*^2^ = 0.95 and the accuracy in terms of RMSE improves to 0.90 kcal mol^−1^. However, performance across force-fields is not consistent: if OPLS and CHARMM are used, different outliers are observed and the correlation with the ZA loop behaviour is not recapitulated, likely reflecting parameterization as a confounding problem. The results provide a benchmark standard for future study and comparison.

## Abstract 2

The enthalpic and entropic components of ligand–protein binding free energy reflect the interactions and dynamics between ligand and protein.

## Graphical abstract

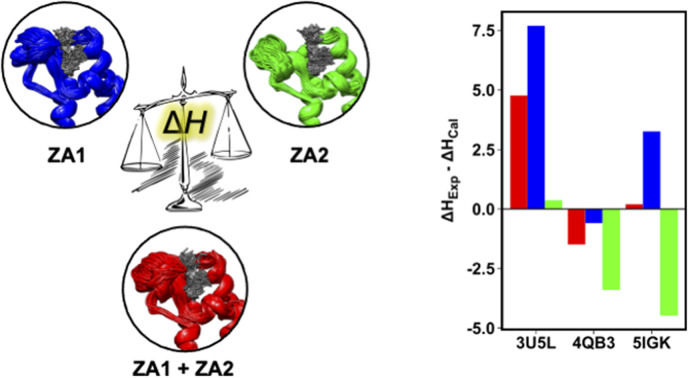

## Introduction

In recent years, there has been significant progress in predicting the binding free energy of small ligands for protein receptors.^1–3^ In contrast there has been relatively little progress in the accurate computation of the underlying thermodynamic components, namely the enthalpy (Δ*H*) and entropy (*T*Δ*S*). Accurate computation of enthalpy has historically been viewed as particularly challenging^4–7^ due to the large fluctuations in potential energy that systems tend to undergo and thus any estimates of the mean value would likely require vast amounts of sampling. Nevertheless, having a reasonably reliable estimation of the enthalpy, and more importantly the error estimate, would be extremely useful in understanding the role of the underlying contributions, especially in the context of drug-design.^8,9^ From a medicinal chemistry point of view, enthalpic contributions are perhaps intuitively easier to understand and conceptualize than the entropic components. During a fragment or lead compound elaboration, where Δ*G* is often being optimized, it would be extremely valuable to know during those steps that the changes made to the compound were indeed giving the expected improvement to the Δ*G via* the designs that the medicinal chemist has suggested. Often, such designs are focused around improving interactions between chemical moieties with the expectation that there is a gain in favourable enthalpy. The ability to compute and confirm this as part of the optimization process would be extremely useful. Moreover, being able to compute enthalpy reliably should provide quantitative insight into the phenomenon of entropy-enthalpy compensation.^10^

The increase in computational power over the past decade has meant that the accurate calculation of enthalpies of binding might soon be realized and indeed work, in particular from the Gilson group on small host–guest and other systems^11–14^ has showed strong potential. However, despite the promising results obtained for small model systems, the calculation of enthalpy contributions for larger proteins has remained challenging and it is not even known what level of performance could be obtained even for well-characterized protein–ligand systems, such as bromodomains.^15,16^

**Fig. 1:**
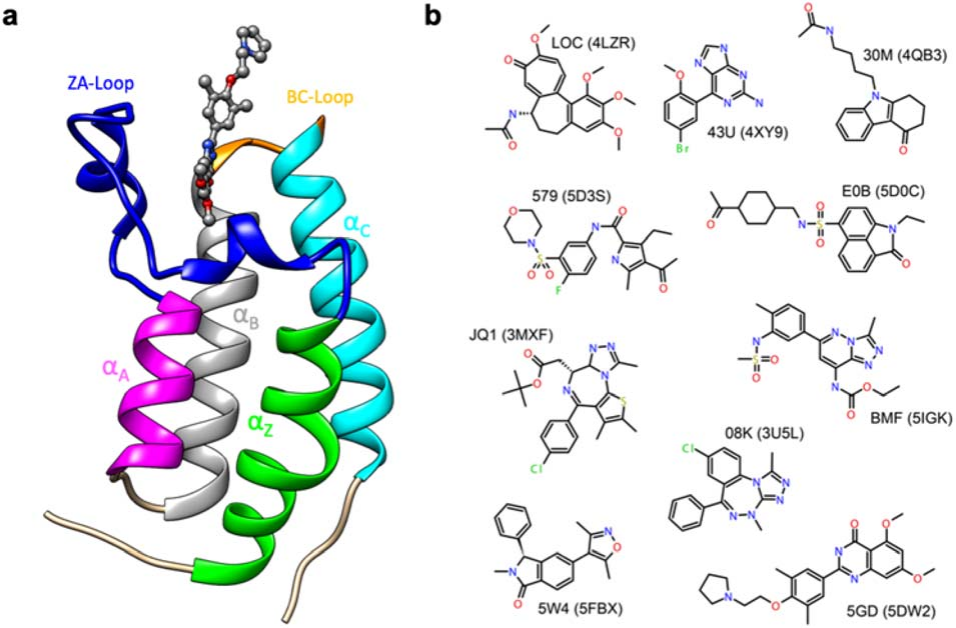
(a) Cartoon of BRD4-1 with ligand complex (PDB:5DW2). (b) Chemical structures of compounds with their three-letter identifiers and corresponding PDB IDs for the complexes.

Thus, in order to evaluate the performance of enthalpy calculations for protein–ligand binding, we assembled a data set based on bromodomains, in a similar vein to that which we had previously done for absolute binding free energy (ABFE) calculations.^15^ Bromodomains (BRDs) are protein–protein interaction modules that selectively recognize acetylated lysine (*K*_ac_) residues as a key event in the epigenetic reading process. A total of 61 human BRDs has been identified in 46 different proteins, which consist of eight protein families.^17^ Despite these many different families, all BRDs have a conserved structure that contains a left-handed bundle of four α helices (α_Z_, α_A_, α_B_, α_C_), linked by loop regions (ZA and BC loops), which surround the *K*_ac_ binding site (Fig. 1a). Among eight families present in human proteome, the bromodomain and extraterminal (BET) family is characterized by two tandem N-terminal BRDs and an extraterminal (ET) domain, and is composed of BRD2, BRD3, BRD4, and BRDT.^18^ BRD4 is thus a representative member of the BET family and has roles in the activation of critical genes involved in cell growth and cell cycle progression.^19^

Given the strong in cell-cycle, it is perhaps not surprising that BRD4 has also been implicated in inflammation and cancer progression resulting in many inhibitor and probe molecules being developed^17^ and indeed this is an ongoing activity. Given the wealth of biophysical and structural data that already exists and the desire to develop yet more probes with improved selectivity, bromodomains represent an ideal test system for computational studies.^15,20–30^ In the vast majority of complexes, the binding pocket does not show major differences in conformation, and this probably contributed to the success of rigorous free energy methods. However, it has recently been suggested the bromodomain fold is quite dynamic, including the relatively recent suggestion of cryptic pockets.^24,29,31^

The first bromodomain of BRD4, BRD4-1 has extensive biophysical and structural data associated with it (Table S1†), and we reasoned this would provide an ideal test case for investigating our current ability to predict enthalpy and what additional insight into binding thermodynamics we might also gain. Here, we perform binding enthalpy calculations using the direct method for a non-redundant set of BRD4-1 and ligand complexes (Fig. 1b).^6,11,14^ Our results show that in the very best case scenario the absolute enthalpy can be calculated for such systems to an error of about 1 kcal mol^−1^. Obtaining results consistent across force-fields remains difficult. However, in some cases, we were able to identify the source of errors in initial outliers and that these can give insight into the well-known problem of enthalpy-entropy compensation (Fig. S1†). In particular, the role of a key loop (the ZA loop) near the binding site is discussed.

## Methods

### Building the benchmark

Initially, all ITC data was collected from the literature, and ITC entries with PDB structures were filtered for the further steps (Table S1†). Ligands in the PDB structures were clustered using binning clustering with default 0.4 similarity cut-off in the ChemMine Tools server (https://chemminetools.ucr.edu/).^32,33^ Representative PDB structures with the best resolution were selected from each cluster for the final benchmark. We use the PDB codes as representing names for all ligands throughout the manuscript for brevity and ease of referral to structures.

### System setup

The initial conformations were taken from crystal structures (3MXF, 3U5L, 4LZR, 4QB3, 4XY9, 5D0C, 5D3S, 5DW2, 5FBX, 5IGK, 2OSS). Missing atoms in the crystals were modeled with the DockPrep tool in UCSF Chimera^34^ and all heteroatoms were removed from the system except the ligand of interest and all crystallographic waters. The N-terminal tail was deleted up to residue Asn54 to reduce computational cost and complexity of the simulations. Terminal residues were patched with acetylated N-terminus and amidated C-terminus using PDB Reader of CHARMM-GUI.^35,36^ Ligand molecules were parameterized with the general AMBER General Force Field for organic molecules (Version 2.11, May 2016)^37^ and AM1-BCC^38,39^ charges using AmberTools19. We used the Amber ff14SB force field for the protein and the TIP3P water model for water molecules.^40,41^ A periodic cubic water box was used for all systems with 20 000 water molecules for the complex and receptor only simulations while 2000 water molecules for used for ligand and solvent-only simulations. The exact same setup was used for the comparison of AMBER to the CHARMM and OPLS Force Fields. Protein topologies were created using the CHARMM36 (ref. 42) and OPLS-AA/M^43^ force fields, while ligands topologies and parameters were generated using the CHARMM General Force Field (CGenFF)^44–46^ and the OPLS/CM1A Parameter Generator for Organic Ligands (LigParGen).^47,48^

### Quantum-optimized parameters

We optimized GAFF2 parameters for 08K(3U5L), 30M(4QB3), BMF(5IGK) and three different ionization states of HEPES using the Psi4 *ab initio* quantum engine at the HF/6-31G* level of theory.^49^ Atomic charges were fitted to reproduce the electrostatic potential (ESP). All steps for getting optimized parameters and charges were performed using the parameterize parameterization tool (https://software.acellera.com/docs/latest/parameterize), which attempts to improve the quality of parameters.^50^ Parameter file are available at doi: https://doi.org/10.5281/zenodo.7534582.

### Absolute binding enthalpy calculations

**Fig. 2:**
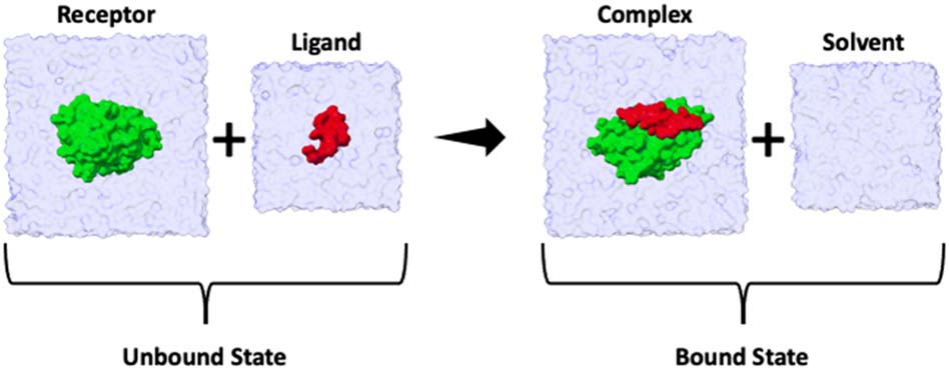
Summary cartoon of the four simulations required to compute the enthalpy of binding for each ligand. Two simulations correspond to the unbound state (where 〈*E*〉_receptor_ and 〈*E*〉_ligand_ are computed) and two simulations to the bound state (where 〈*E*〉_complex_ and 〈*E*〉_solvent_ are computed). The overall enthalpy can then be computed *via*eqn (1).

The binding enthalpy (Δ*H*) is calculated by computing the terms in eqn (1), where 〈*E*〉_complex_, 〈*E*〉_solvent_, 〈*E*〉_receptor_, and 〈*E*〉_ligand_ are the averaged potential energies of the system as computed *via* four separate simulations (Fig. 2). In this method, the number of atoms between the bound and unbound state of the complex should exactly balance. To achieve this, we first solvate the simulation system and then delete the excess water molecules to balance the bound and unbound states. Note that the pressure–volume contribution for the binding enthalpy is negligible.^11^

1 Δ*H* = 〈*E*〉_complex_ + 〈*E*〉_solvent_ − 〈*E*〉_receptor_ − 〈*E*〉_ligand_

All simulations were performed using the GROMACS v2020.3 software package.^51–54^ A 3-step steepest descent energy minimization with a maximum force of 10 kJ mol^−1^ nm^−2^ was applied to all systems. In the first step, position restraints with an harmonic potential with a force constant of 1000 kJ (mol^−1^ nm^−2^) were applied for all heavy atoms, then there remove for solute heavy atoms, and the final step removed all restraints. NVT and NPT ensemble simulations for 1 ns were performed to equilibrate all systems with position restraints with the harmonic potential at a force constant of 1000 kJ (mol^−1^ nm^−2^) on heavy atoms of protein and ligand. Additionally, another NPT ensemble simulations for 1 ns was performed without restraints before the production run for data collection. The V-rescale and Parrinello–Rahman algorithms equilibrated the temperature at 300 K and the pressure at 1.0 bar, respectively. The leap-frog algorithm was used to run 20 independent 100 ns MD simulations with 2 fs time step. All input files for all simulations using AMBER, CHARMM and OPLS FF are available at doi: https://doi.org/10.5281/zenodo.7534582.

The average of the potential energy and the estimate of the standard error were calculated by performing re-blocking analysis using the pyblock tool (https://pyblock.readthedocs.io)^55^ for all individual calculations. In all cases blocks were used that led to the maximum standard error of the mean (SEM).

### Absolute binding free energy calculations

Absolute binding free energy calculations were performed using conformations obtained from the binding enthalpy simulations. For six complexes (3U5L, 4LZR, 4QB3, 4XY9, 5DW2 and 5IGK), we performed two sets of simulations reflecting the different conformations of the ZA loop. MDRestraintsGenerator, which is a framework for generating restraints for MD simulations was used to provide the optimal Boresch restraints (1 distance, 2 angles and 3 dihedral harmonic restraints) (https://github.com/IAlibay/MDRestraintsGenerator). The ligand nonbounded interactions were decoupled using a linear alchemical pathway for the van der Waals and the coulombic transformations with Δ*λ* = 0.05 and 0.1, respectively. The ligand restraints transformation had 12 non-equally distributed *λ* values (0.0, 0.01, 0.025, 0.05, 0.075, 0.1, 0.15, 0.2, 0.35, 0.5, 0.75, 1.0). Each calculation for absolute binding free energy comprised a total of 44 windows for the complex simulations and 32 windows for the ligand simulations. Each window was completed with 5 step runs. Firstly, energy minimization was carried out using the steepest descent algorithm with 10 000 steps. Then, 10 ps NVT ensemble was performed using leap-frog stochastic dynamics integrator with harmonic position restraints on the solute heavy atoms with a force constant of 1000 kJ mol^−1^ nm^−2^. After that, 100 ps isotropic ensemble using the Berendsen coupling algorithm was run with the same position restraints. Moreover, another NPT ensemble with the Parrinello–Rahman coupling algorithm was performed for 100 ps without position restraints. Finally, 10 ns production runs were performed for data collection.

### Constructing the unit cell

A crystal unit cell for 2OSS was built to investigate the effect of crystal packing on the ZA-loop conformation. The Cell Unit tool in UCSF Chimera was used for constructing the unit cell for 2OSS with the *P*2_1_2_1_2_1_ space group. Missing atoms in the crystals were modeled with the DockPrep tool in UCSF Chimera and 1,2-ethanediol molecules were removed from the system while crystallographic waters were kept. The cell unit contains 4 chains with lengths 37.418, 44.139, 78.413 on the xyz dimensions. We used three different force field including Amber FF14SB,^40^ CHARMM36,^56^ and OPLS-AA/M^57^ force field. Simulations were performed with 3 replicates for the crystal lattice and 12 replicates for the single chains.

### Preparing figures

All plots were prepared by using ggplot2 which is an open-source data visualization package for the R programming language.^58^ Visualization and analysis of molecular structures were made with UCSF Chimera.^34^

## Results

### Collating a non-redundant BRD4-1 benchmark data set

For this study, we first collected all binding data with thermodynamic components derived from ITC for BRD4-1 from the literature. Then only data sets that could be linked to high-resolution (better than 2.0 Å) were retained. Given the interest in generating probes and drug-molecules against BRD4-1, many studies report lead-optimization attempts^59,60^ and this leads to many similar ligand molecules within the data set and a significant issue of redundancy. To obtain a non-redundant dataset, ligand molecules were clustered using binning with a 0.4 similarity cut-off with the ChemMine Tools server.^32,33^ After clustering, we had 14 different clusters with cluster sizes ranging from 1 to 11 (Table S1†). Four clusters, each with a single protein (4OGI, 4OGJ, 5EGU, 5BT4), were filtered out since interactions between two protein chains in the crystal lattice were bridged by the ligand and this was deemed artificial. Then, representative PDB structures with the best resolution were selected from each remaining clusters for the final benchmark (Table 1 and Fig. 1b).

**Table 1:** Non-redundant BRD4(1) benchmark. All values are in kcal mol^−1^

PDB ID	Resolution (Å)	Space group	Ligand ID	Δ*H*	*T*Δ*S*	Δ*G*	Reference
3MXF	1.60	*P*2_1_2_1_2_1_	JQ1	−8.42	1.22	−9.64	Filippakopoulos *et al.*^61^
3U5L	1.39	*P*2_1_2_1_2_1_	08K	−6.16	2.00	−8.16	Filippakopoulos *et al.*^17^
4LZR	1.85	*P*2_1_2_1_2_1_	LOC	−9.00	−2.60	−6.40	Lucas *et al.*^62^
4QB3	0.94	*P*2_1_2_1_2_1_	30M	−6.62	0.93	−7.55	Gacias *et al.*^63^
4XY9	1.83	*P*2_1_2_1_2_1_	43U	−6.09	0.94	−7.03	Picaud *et al.*^64^
5D0C	1.49	*P*2_1_2_1_2_1_	E0B	−10.20	−2.52	−7.68	Xue *et al.*^65^
5D3S	1.75	*P*2_1_2_1_2_1_	579	−9.77	−1.73	−8.04	Hügle *et al.*^66^
5DW2	1.12	*P*2_1_2_1_2_1_	5GD	−10.10	2.10	−8.00	Raux *et al.*^67^
5FBX	1.85	*P*2_1_2_1_2_1_	5W4	−15.57	−4.66	−10.90	Montenegro *et al.*^68^
5IGK	1.70	*P*2_1_2_1_2_1_	BMF	−11.09	−1.36	−9.73	Picaud *et al.*^69^

### Absolute binding enthalpy calculations

Relative binding enthalpies have been reported for protein–ligand systems using the direct method.^6,14^ Absolute calculation of binding enthalpy using the direct method requires a set of four simulations including bound and unbound states of the system (see Methods). In assessing binding enthalpies, sampling all conformational space is the key factor to achieve sufficient convergence of the potential energy. In previous studies, Roy *et al.* performed 40 independent 10 ns simulations to get sufficient sampling,^6^ moreover, Li and Gilson reached over 250 μs simulations by seeding every 200 ns block with a new random number for the relative binding enthalpy calculation of a protein–ligand system.^14^ Here, we performed 20 completely independent repeats of 100 ns simulations for each system. To assess convergence, we employed the blocking method^55^ where the enthalpy is averaged over successively larger blocks and for each block size the standard error of the mean is computed. As discussed by Henriksen *et al.*,^13^ in an ideal case the SEM will display a plateau, but this is not always the case and it is not easy to automate detection of such a plateau either. Therefore, again following the work of Henriksen *et al.*^13^ we took the maximum SEM value (Fig. S2 and Table S2†) to err on the side of caution. For most complexes the maximum SEM is ∼0.6 kcal mol^−1^ but even the maximum (for 5FBX) is ∼1.1 kcal mol^−1^. As expected, the ligand and solvent only profiles converge earlier than the complex.

**Fig. 3:**
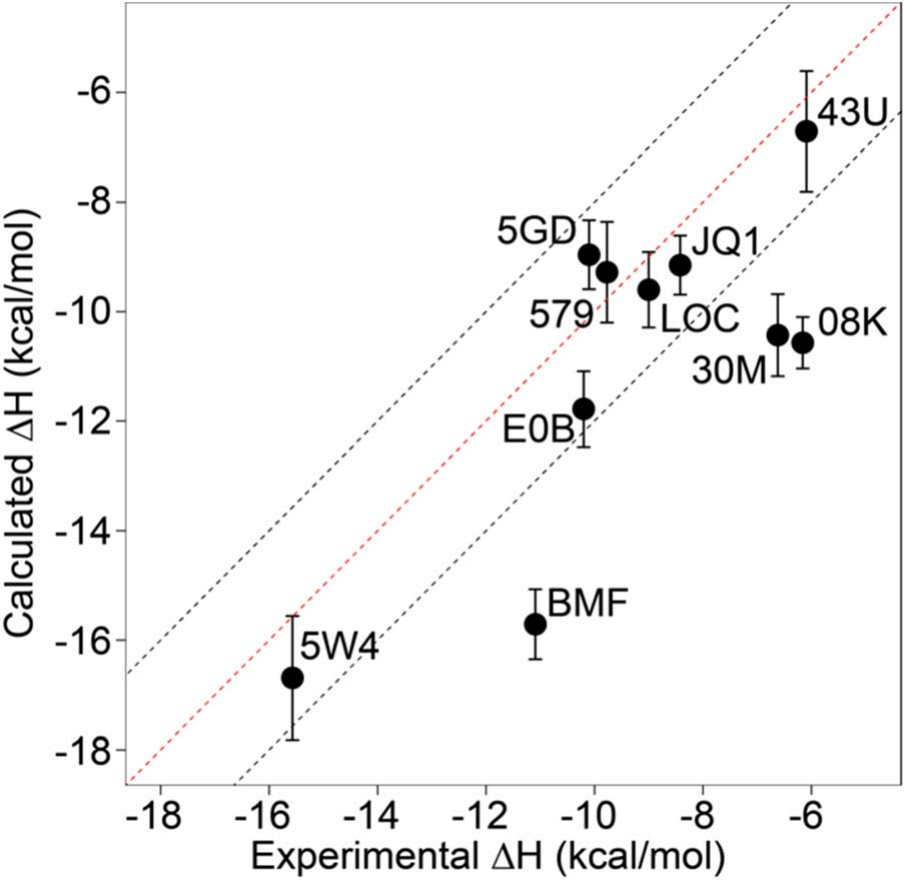
Comparison of calculated binding enthalpies from experimental values. Error bars show SEM of the mean. The line of equivalence is shown in red and the black dashed lines indicate the 2 kcal mol^−1^ error limit. Three letter codes are the ligand codes as in Table 1 and Fig. 1.

From these initial simulations with AMBER, the correlation with experiment was moderately good with an *R*^2^ = 0.60, and an average of Kendall's *τ* = 0.42. The accuracy of the calculation is perhaps surprisingly good with an average of root-mean-square error (RMSE) = 2.49 kcal mol^−1^. Most of the binding enthalpies are within 2 kcal mol^−1^ absolute difference of experimental values (Fig. 3 & Table S2†). The best binding enthalpy predictions were obtained for 5D3S with the XD44 (4-acetyl-3-ethyl-*N*-[4-fluoro-3-(morpholin-4-ylsulfonyl)phenyl]-5-methyl-1*H*-pyrrole-2-carboxamide) ligand (579) and 4LZR bound to colchicine (LOC).

Conversely, 3U5L in complex with a benzo-triazepine ligand (08K), 4QB3 with olinone (30M) and 5IGK with bromosporine (BMF) provided the worst binding enthalpy predictions as outliers (Fig. 3 & Table S2†).

Although some of the computed enthalpy values are in excellent agreement with the experimental data, there are some clear outliers (08K, 30M and BMF), all of which are overestimates. We thus then sought to investigate these further.

We first considered improved parameterization. We optimized ligand charges for the outliers using the Psi4 *ab initio* quantum engine with the HF/6-31G* level of theory. After optimization, there was good agreement of the energy profile of GAFF2 parameters with the reference quantum mechanics (QM) calculations (Fig. S3†). Then, all simulations with 20 replicates, with a total of 120 simulations, were rerun using the optimized parameters for 3UL5, 4BQ3, and 5IGK. The binding enthalpy calculation for 4QB3 was improved at −5.13 ± 0.72 kcal mol^−1^, which is closer to the experimental value and within 2 kcal mol^−1^. However, both 3UL5 and 5IGK still remained as outliers. The predicted binding enthalpy for 5IGK with bromosporine was slightly improved, but for 3UL5 the values actually got worse and increased the absolute difference to 7.69 kcal mol^−1^ from the experimental value (Table S3†). Thus, re-parameterization could not account for all of the poor performance.

We next considered the role of the buffer. Thus far, we have performed all simulations with pure water to reduce complexity, whereas the ITC experiments were mostly performed in 50 mM HEPES and 150 mM NaCl solution (Table S1†). However, binding thermodynamics may be sensitive to the solvent composition for both experimental^70,71^ and computational^12,16^ studies. To investigate the role of the buffer and in particular to what extent replicating the conditions of the experiments influenced the calculations, we set up simulations having three different ionization states of HEPES (Fig. S4†) and NaCl for the apo-receptor (2OSS) and the 3U5L and 5IGK complexes. Force field parameters of HEPES were obtained using Psi4 with the HF/6-31G* level of theory while Na^+^ and Cl^−^ parameters were used as provided in the Amber ff14SB force field. We then performed further simulations with 20 replicates with a total of 60 simulations of the apo-receptor (2OSS), 3U5L and 5IGK in 50 mM HEPES and 150 mM NaCl solution. Although we obtained sufficient convergence of potential energy for these simulations (data not shown), the uncertainty goes up, as expected, because HEPES and NaCl make the system more complex, and this requires longer simulations or more replicas. However, the accuracy of enthalpy prediction itself remained poor. The enthalpy for 3U5L was slightly improved but 5IGK gave a worse result than previous calculations (Table S3†). Thus, we concluded that explicit treatment of buffer in the calculations was not the main reason for large deviations from experimental data.

### The ZA-loop adopts an alternative conformation that strongly affects binding enthalpy

**Fig. 4:**
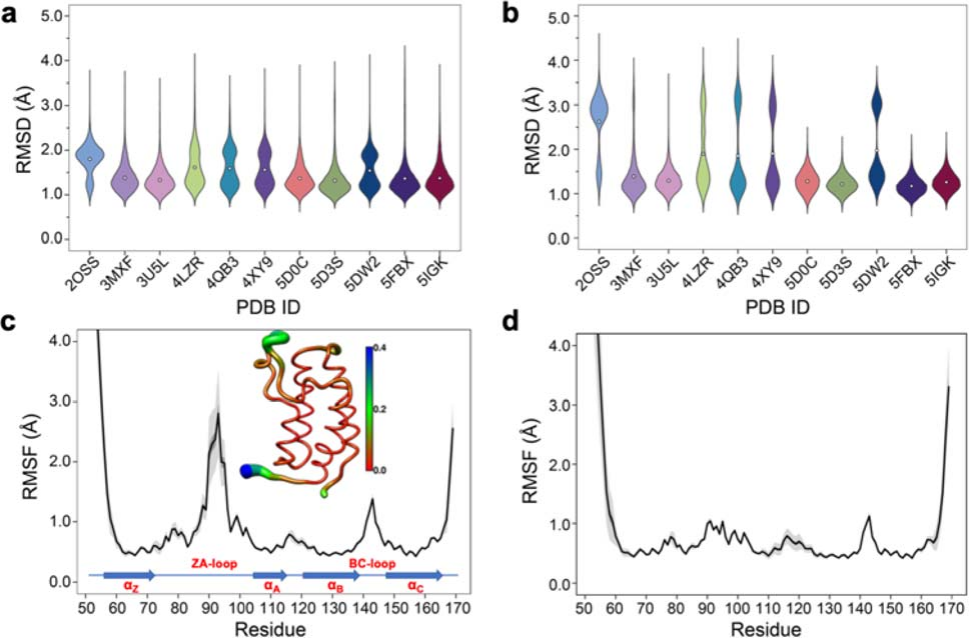
(a) RMSD violin plots for backbone atoms of 2OSS apo-receptor and complex simulations, white circle represents overall mean value of all 20 repeats. (b) RMSD violin plots for ZA-loop (76–106 residues – see Fig. 1) backbone atoms of 2OSS apo-receptor and complex simulations after fitting whole protein backbone. (c) RMSF for backbone atoms of 2OSS apo-receptor. The inset figure shows regional fluctuations on the BRD4-1. (d) RMSF for backbone atoms of 5IGK complex simulations. Black line gives average RMSF of 20 repeats while grey shade represents standard deviation.

Simple observation of trajectories revealed a significant structural deviation in the ZA loop of some simulations, especially the apo structure, 2OSS (Fig. 4a–c and 1a). Whilst some ligands appear to stabilize the ZA-loop in the crystal-like conformation (5IGK, 5FBX, 5D3S, 5D0C, and 3U5L simulations), it is clear that the others afford the ZA loop a greater level of dynamics as evidenced by simple root-mean squared fluctuations (RMSF – Fig. 4b). Closer inspection revealed that in fact the ZA-loop can move to a distinct and alternative conformation, which in the case of the apo (2OSS) state, exists for approximately 75% of the 2 μs simulation time (Fig. 4c). In this alternative conformation, the ZA-loop moves outwards away from the acetyl-lysine binding pocket and induces a short helix (residue 88–91) within the ZA-loop. This outward movement makes the binding pocket open and more accessible. To investigate the role of this loop behaviour on the enthalpy we extended the number of repeats of the apo state to 100, to ensure that we obtained sufficient sampling of the crystal-like conformation of the ZA-loop. For the remainder of the discussion, we refer to the crystal-like conformation of the ZA-loop as ZA1 while the alternative ZA-loop conformation as ZA2.

After completing 100 replicates of the apo-receptor (a total of 10 μs of simulation), we checked the difference in potential energy between the ZA1 and ZA2 conformational states, which was 0.84 ± 0.2 kcal mol^−1^. Previous work has reported the barrier between these states as being of the order of ∼2 kcal mol^−1^.^26^ In addition to the apo-receptor (2OSS) displaying this alternative ZA2 loop conformation, so did some ligand-bound simulations including 4LZR, 4QB3, 4XY9, and 5DW2 (Fig. 4b and S5†). We then also checked the difference of the potential energy between ZA1 and ZA2 (Table S4†) for these complexes. The 4LZR complex gave the biggest difference of the potential energy with 5.77 ± 1.5 kcal mol^−1^ while 4XY9, at 1.83 ± 1.93 kcal mol^−1^, had the lowest difference amongst these four complexes. Of these four complexes, only 5DW2 exhibited lower potential energy for the ZA2 conformation.

**Fig. 5:**
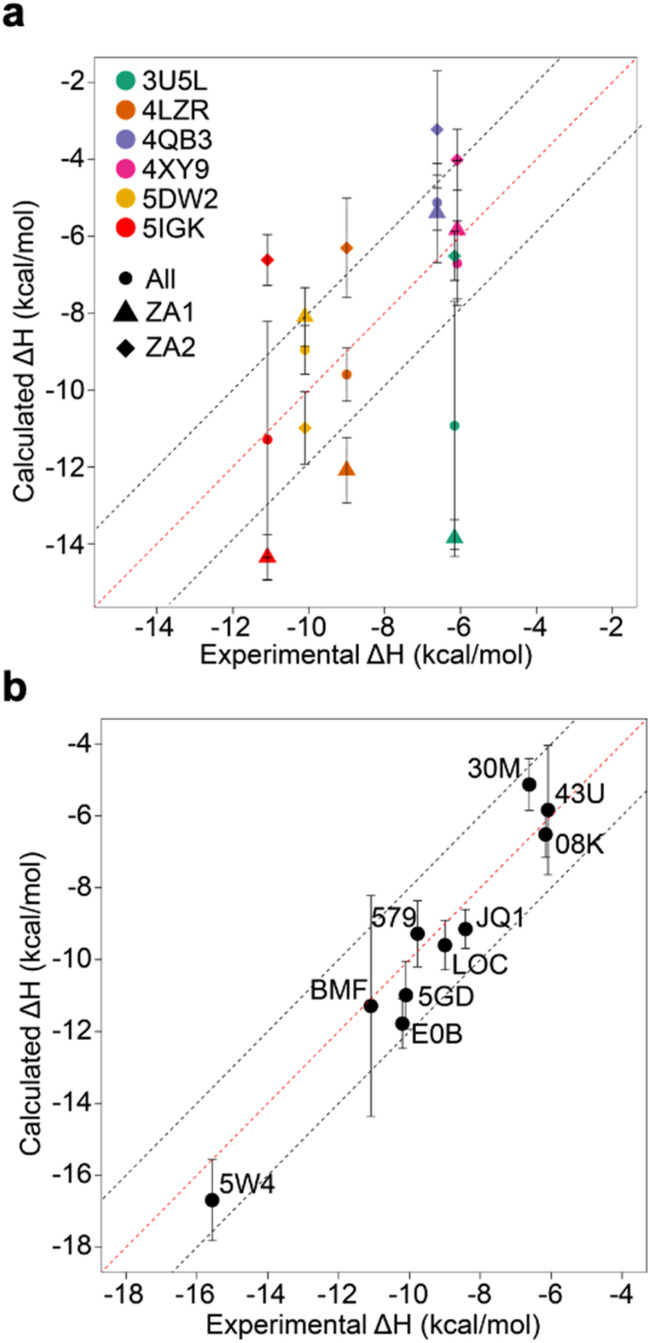
Comparison of calculated binding enthalpies to experimental values. (a) Values obtained by considering only ZA1 (triangles) or ZA2 (diamonds) or all (circles) ZA loop conformations for outliers and complexes observed to adopt alternative conformations of the ZA loop. (b) The best calculated binding enthalpy values that can be obtained for the whole dataset. Note that for 4X9Y the “All” data point does not sit between the ZA1 and ZA2 points as might be intuitively expected – this is due to the nature of the blocking analysis where we use blocks that give the largest SEM. Error bars show the maximum standard error the mean estimate.

The existence of these significant differences of potential energy thus raised the question of how these two conformations affect binding enthalpy calculations. To explore this, we calculated binding enthalpies using ZA1 or ZA2 conformations exclusively for the 4LZR, 4QB3, 4XY9, and 5DW2 complexes (Fig. 5 & Tables S4 and S5† – note strictly speaking the apo states of ZA1 and ZA2 should be considered but in practice the 0.84 kcal mol^−1^ difference in Δ*H* between these two conformations in the apo state was within error). Using exclusively ZA1 or ZA2 conformations for 4LZR and 4XY9 gave less accurate predictions compared to use of both conformations (Fig. 5). 4QB3 gives a more accurate enthalpy prediction with ZA1 than with ZA2 alone or use of all simulation data combined. This also explains why the simulations with QM-refined charges (Table S3 and Fig. S3†) and gave more precise results than the initial runs, since occupation of the ZA1 conformation in the second simulation set was more than the first one (Table S5†). Conversely, 5DW2 interestingly gave more accurate binding enthalpy estimates when only the ZA2 conformation was used.

As the remaining outliers, 5IGK and 3U5L, were comprised solely of ZA1 conformations in all 60 replicates and given the above indication of the importance of the ZA loop behaviour, we decided to initiate replicates for these two complexes starting from the ZA2 conformation. For this purpose, we extracted a snapshot having the ZA2 conformation from a random apo-receptor simulation, then we manually docked BzT-7 (08K) of 3U5L and bromosporine (BMF) of 5IGK to the receptor *via* superimposition and performed 20 repeats of 100 ns. The ZA-loop stayed as the ZA2 conformation in all simulations for 3U5L (Fig. S9†) and almost all simulations for 5IGK (there was a transition from ZA2 to ZA1 in the last 20 ns of only one simulation). We obtained sufficient convergence of potential energy for these simulations, then binding enthalpies were calculated. Surprisingly, 3U5L gave a highly accurate prediction with −6.52 ± 0.64 kcal mol^−1^, which is 0.36 kcal mol^−1^ away from the experimental value (−6.16). However, we did not obtain an improvement in accuracy of enthalpy for 5IGK with ZA2, indeed the experimental value is nearly in the middle of the predicted binding enthalpies for ZA1 and ZA2, suggesting that both conformational states contribute to the enthalpy. To confirm this, we combined simulation data for both ZA1 and ZA2 from 40 simulations in total and recomputed the binding enthalpy. The combined simulation data gave a prediction of −11.29 ± 3.07 kcal mol^−1^, which is 0.20 kcal mol^−1^ away from the experimental value (−11.09 kcal mol^−1^) (Fig. 5a). Taken together these computations reveal a hidden complexity of binding thermodynamics in what might be considered a relatively simple system. When the best predictions are all combined (Fig. 5b), an *R*^2^ of 0.95 and an RMSE of 0.90 kcal mol^−1^ can be obtained, and although this was arrived at retrospectively and in dependent on known observations, it does at least illustrate that “chemical accuracy” (∼1 kcal mol^−1^) of these calculations is within reach.

### The relationship to the absolute binding free energy

Given the influence of the ZA loop conformation on the enthalpy predictions, we were interested to see to what extent the ZA loop conformation also affected the absolute binding free energy, Δ*G*. Thus, for the four complexes that displayed alternative ZA loop conformations (see Fig. 5a); 4LZR, 4QB3, 4XY9 and 5DW2, along with the two outliers 5IGK and 3U5L (where loop stability is greater for ZA1 Fig. S7†), we computed the binding free energies (Table 2). For four of the complexes (4LZR, 4QB3, 4ZY9 and 5DW2) the loop conformation that favours the lower Δ*G* is mirrored by the enthalpy results (Table S4†). The 3U5L complex however, is a more complicated result. Whereas the enthalpy calculations suggest that the ZA2 conformation leads to better agreement with experiment (Fig. 5a), the free energy calculations give a lower binding free energy for the ZA1 conformation (albeit heavily overestimated compared with experiment). Similar trends for 3U5L have been reported by Heinzelmann *et al.*^26^ using a different approach, the Attach-Pull-Release method, which produced an over-estimated result (−10.61 kcal mol^−1^) for ZA1 but a closer-to-experiment result (−8.07 kcal mol^−1^) for ZA2. Furthermore, an almost similar result (−9.9 ± 0.8) for ZA1 has been observed by Aldeghi *et al.*^15^ and also by Bertin^72^ (−9.1 ± 0.3) (https://thesis.unipd.it/handle/20.500.12608/21280) using the alchemical decoupling free energy method. Together, these results suggest that the 08K ligand in 3U5L tends to give an overestimated binding affinity when the crystallographic conformation (ZA1) is used. Compared with other ligands that bind BRD4(1), the ligand has modest enthalpic contributions to the binding free energy, but one of the most favourable entropic contributions (Table 1). Analysis of the energetic components of the enthalpy (Table S6†) shows that there is a large coulombic contribution in the ZA1 conformation that is almost completely absent in ZA2. In the case of 5IGK (bromosporine complex) the Δ*G* values is higher than experiment for the ZA1 loop conformation but lower for the ZA2 conformation. The value obtained for the ZA1 conformation in this work is completely consistent with the value we obtained in previous work^21^ (and was initiated from a dock to the apo state, thus using the ZA1 conformation). Calculated Δ*G* and Δ*H* values for both ZA loop conformations are nearly equidistant to the experimental values for 5IGK.

**Table 2:** ABFE results for ZA loop conformations^a^

PDB ID	Δ*G*_Exp_	Δ*G*_ZA1_	Δ*G*_ZA2_
3U5L	−8.16	−11.36 ± 0.28	−6.85 ± 0.28
4LZR	−6.40	−6.52 ± 0.53	−2.50 ± 0.21
4QB3	−7.55	−7.55 ± 0.37	−4.94 ± 0.64
4XY9	−7.03	−4.88 ± 0.48	−2.87 ± 0.95
5DW2	−8.00	−7.69 ± 0.69	−8.10 ± 0.32
5IGK	−9.73	−11.79 ± 0.37	−4.84 ± 0.40

a Δ*G* values were obtained *via* running 3 independent ABFE calculation using different starting structures.

### The dynamics of the ZA loop

**Fig. 6:**
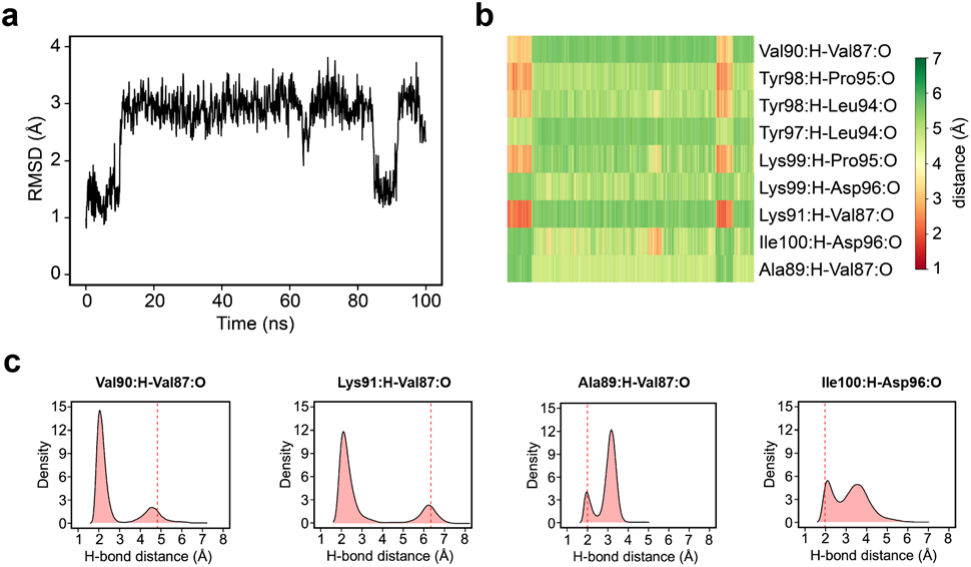
(a) RMSD plot for ZA-loop (76–106 residues) backbone atoms from a representative case for the transition between ZA1 and ZA2 from one apo-receptor simulation of 100 replicates. (b) Hydrogen bond distance profile of some important backbone hydrogen bonds in the ZA-loop from the representative simulation. (c) Density plots of distance distribution for key hydrogen bonds across 100 replicates of apo-receptor simulations. The red dashed line shows the average distance of starting minimized structures from ZA1 conformations.

Given the clear role of the ZA-loop conformations in enthalpy prediction accuracy, we analysed the 100 apo-receptor simulations in terms of the transition between ZA1 and ZA2. The transition from ZA1 to ZA2 occurs in all simulations with a mean transition time of 22.59 ± 1.84 ns. Interestingly, the transition was in most cases irreversible and the reverse transition from ZA2 to ZA1 happened in only two simulations. Moreover, the ZA-loop quickly transitioned back again to ZA2 whenever a reverse transition happened. Heinzelmann *et al.* reported ZA2 to be more favourable than the ZA1 by −2.54 kcal mol^−1^.^26^Fig. 6a shows an example of the reverse transition from ZA2 to ZA1 around 80–90 ns. A pairwise RMSD analysis also confirmed the reverse transition from ZA2 to ZA1 in the simulation (Fig. S6†) and is clearly obvious by simple observation (Movie M1†).

**Fig. 7:**
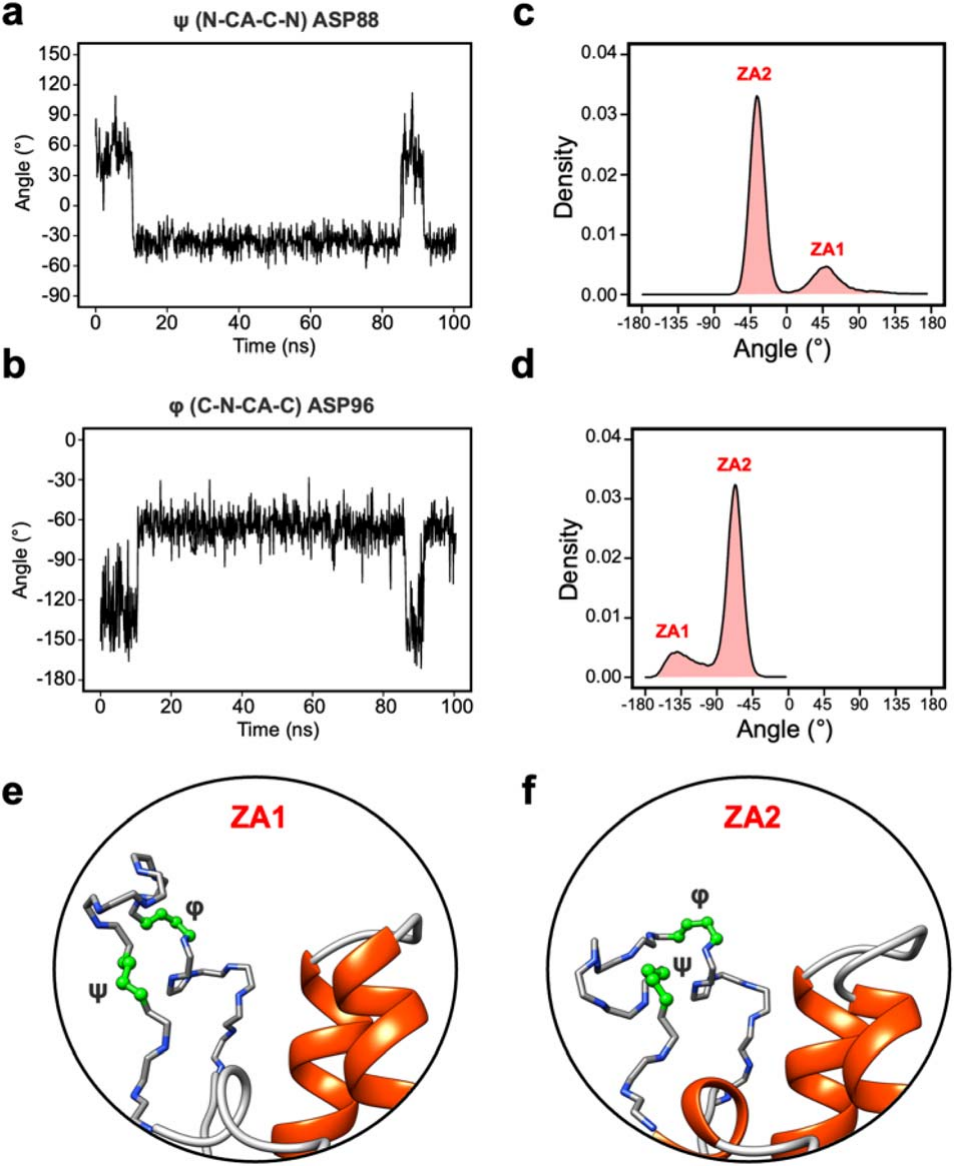
(a) The angle change of the *ψ* (N-CA-C-N) Asp88 from the simulation in Fig. 5a. (b) The angle change of the *φ* (C-N-CA-C) Asp96 from the simulation in Fig. 5a. (c) The angle distribution of the *ψ* (N-CA-C-N) Asp88 across 100 apo-receptor simulations. (d) The angle distribution of the *φ* (C-N-CA-C) Asp96 across 100 apo-receptor simulations. (e and f) Main chain representation of ZA1 and ZA2 conformations. Green ball & stick regions show *φ* and *ψ* dihedral angles.

During the transition, the hydrogen bond profiles of key backbone residues change dramatically (Fig. 6b and c) as well as sidechains. The transition of ZA1 to ZA2 is associated with the backbone torsion angles of *ψ* (N-CA-C-N) Asp88 and *φ* (C-N-CA-C) Asp96 (Fig. 7). These two dihedrals behave like a hinge allowing the ZA-loop to transit from ZA1 and ZA2. The *ψ* Asp88 shuttles from 50 to −40° while the *φ* Asp96 moves from −150 to −60°. The distributions of the other backbone torsions in the ZA-loop do not show such clear-cut modal distributions, except *ω* (CA-C-N-CA) of Gln84 but is possibly not related to the transition from ZA1 and ZA2 (Fig. S8†). Potentially, *ω* GLN84 is related to a recently explained hidden transient state of the ZA-loop, where the event includes the breaking of two backbone hydrogen bonds between the ZA-loop and the α_A_ helix.^29^

### Crystal-packing of apo-BRD4-1 explains ZA-loop conformations

**Fig. 8:**
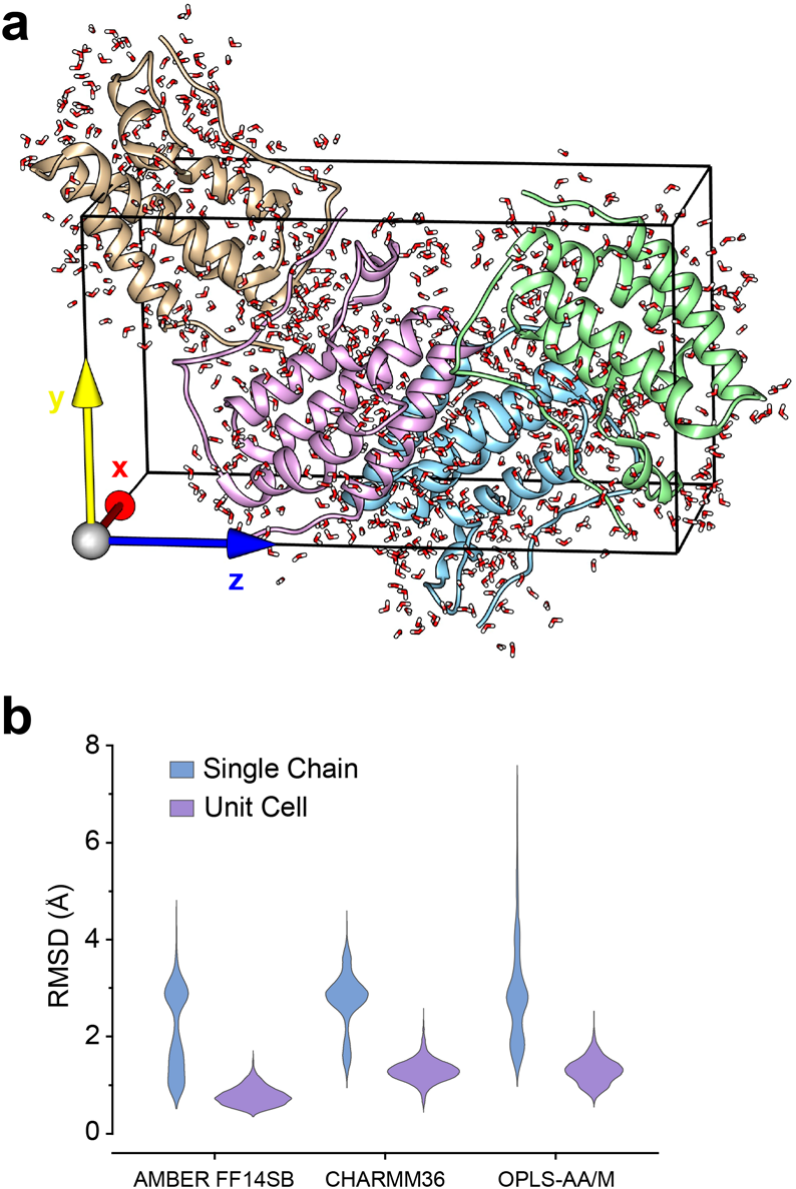
(a) 2OSS crystal cell unit containing 4 identical chains. (b) RMSD violin plots for ZA-loop (76–106 residues) backbone atoms. Blue violin shows ZA-loop RMSD from 12 independent single-chain simulations while purple violins show for the unit cell simulations.

Given the ubiquity of the ZA2 conformation in our simulation data, but lack of observation in crystallographic data we next investigated the role of crystal lattice packing. We built a crystal unit cell for the apo BRD4-1 receptor (2OSS) with the *P*2_1_2_1_2_1_ space group (Fig. 8a). The cell unit contains 4 chains with lengths 37.418, 44.139, 78.413 Å on the *x*, *y* and *z* dimensions. We performed 3 replicates (100 ns) for the crystal unit and 12 replicates (100 ns) for simulations with a single chain (monomer).

We also explored potential force-field influence, by examining two additional force fields: CHARMM36 and OPLS-AA/M. As a result, in total, we performed a total of 9 simulations for a complete unit cell and 36 simulations for a single chain. We were first interested in investigating the dynamics of ZA-loop in both simulation setups. As expected, all chains retained their crystal-like conformation in the unit cell simulations (Fig. 8b). In contrast, in all single-chain simulations in all three different force-fields, the ZA-loop exhibited much higher flexibility (Fig. 8b), thus supporting the notion that crystal-packing artefacts likely constrain the ZA conformation in the apo state. Crystal-packing effects are likely to be present in the complexes as well – simulations of similar unit-cell simulations of the complexes (Fig. S9†) reveal the ZA-loop does not move away from its lattice conformation. Almost all complex PDBs except 5D0C share same space group with the apo BRD4-1 receptor (2OSS) (see Table 1). Interestingly, simulations of the 5D0C lattice appear to allow more flexibility of the ZA-loop.

### Role of force fields in general

**Fig. 9:**
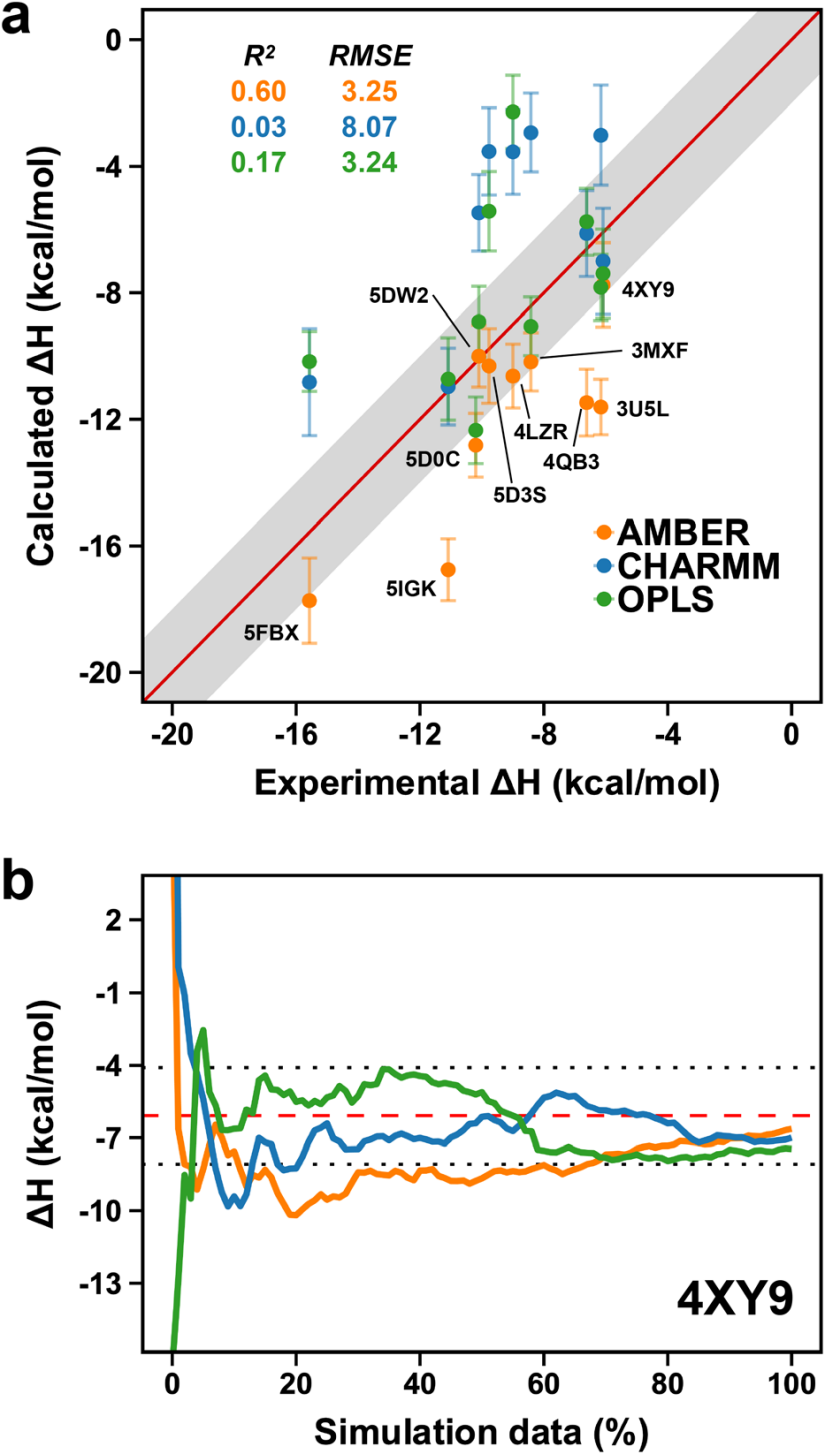
(a) Comparison of AMBER (orange points) to CHARMM (blue dots) and OPLS (green dots). Labels are only shown on the AMBER data points. Error bars show the maximum standard error the mean estimate. (b) Convergence pattern of the calculated Δ*H* for 4XY9 with the three force fields (AMBER, orange; CHARMM, blue and OPLS, green). The red dashed-line is the experimental Δ*H* and the dotted-lines indicate the 2 kcal mol^−1^ error limit.

In the previous section, we investigated the potential influence of force fields on the flexible nature of the ZA-loop and showed the force-fields behaved similarly. We thus decided to extend this approach further and evaluated the performance of different force-fields for computing the absolute binding enthalpies. Our calculations revealed that OPLS and AMBER showed comparable accuracy, whilst the RMSE of the CHARMM calculations was 8.07 kcal mol^−1^ (Fig. 9a). However, it is worth noting that while AMBER produced overestimated values (more negative), it had a higher correlation with experimental values than OPLS and CHARMM. We did not consider the QM optimization and the ZA loop effect in AMBER simulations to ensure a fair comparison in Fig. 9. Among the PDBs evaluated, only 4XY9 showed good agreement with experimental values for all force field (Fig. 9b and Table S7†).

The large error associated with the CHARMM calculations can be attributed to various things. For example, our results indicate that 3MXF and 5D0C with AMBER and OPLS simulations with stable ZA loops in the crystal-like conformation (ZA1) provided more accurate binding enthalpy (Fig. S11 and Table S7†). In contrast, within the CHARMM simulations, the ZA loop exhibited greater flexibility resulting in inaccurate binding enthalpy for 3MXF and 5D0C. We identified 10 and 9 simulations for 3MXF and 5D0C respectively, where the ligand completely left the binding pocket in these simulations, suggesting that default ligand parameterization within the CHARMM system may require additional optimization. Indeed, all but one ligand likely require some addition parameterization as suggested by the CGENFF server (Table S8†).

3U5L with AMBER provided an accurate enthalpy estimate with only the ZA2 conformation, while 5IGK required a combination of ZA1 and ZA2 to obtain a good prediction of enthalpy in AMBER simulations (Fig. 5a). To validate these results with CHARMM and OPLS, we also ran simulations using starting structures with both ZA1 and ZA2 for 3U5L and 5IGK (Fig. S12 and S13†). The ZA loop remained very stable when simulations were started from ZA2 in all cases (Fig. S12†). We observed that the ZA loop of 3U5L remained stable in the ZA1 conformation with AMBER and CHARMM, while it also interestingly transitioned to the ZA2 conformation with OPLS, yielding an accurate result for the binding enthalpy (Fig. S12 and Table S9†).

As CHARMM gave many outliers, we restricted further outlier analysis to OPLS. The OPLS simulations had three distinct outliers (4LZR, 5D3S, and 5FBX) that were not only different from AMBER but also all underpredictions. Whereas for the AMBER outliers there appeared to be a link between the ZA loop conformation and the quality of the prediction, for the OPLS outliers we could not find such a correspondence.

Firstly, 4LZR yielded accurate results with the AMBER forcefield, and the simulations displayed a transition to the ZA2 conformation. In the AMBER simulations, the ZA2 conformation had an occupancy of 26.94%, while in OPLS, it was only 4.41%. Given our previous observations, we suspected that the lower occupancy of ZA2 in OPLS simulations might be responsible for the inaccurate binding enthalpy predictions. We thus conducted six additional simulations explicitly starting with the ZA2 conformation. However, these additional simulations did not result in any improvement in the outcome. We did, however, observe that the ligand in the OPLS simulations appeared to have more flexibility and indeed tended to move away from the binding pocket. Thus, in this case some refinement of the OPLS ligand parameters might well be necessary.

For 5D3S, both AMBER and OPLS displayed a similar occupancy profile for the ZA2 conformation. However, OPLS introduced greater flexibility to the ZA loop compared to AMBER. To investigate the relationship between the ZA loop dynamics and the binding enthalpy, we calculated the binding enthalpies individually for each complex simulation. Surprisingly, seven simulations provided accurate results within a 2 kcal mol^−1^ error limit. These seven simulations exhibited a much more stable ZA loop compared to the remaining simulations and yielded binding enthalpy results similar to those obtained with AMBER (Fig. S14†). Thus, although it is difficult to address why the ZA loop exhibits greater dynamics with OPLS, it does at least support the overall theme that the ZA loop behaviour is important in enthalpy predictions.

Finally, for 5FBX, although the ZA loop exhibited similar behaviour in OPLS as in AMBER, the loop was highly flexible in all simulations. We explored this by additional simulations with dihedral restraints on the *ψ* (N-CA-C-N) of Asp88 and *φ* (C-N-CA-C) of Asp96 to maintain stability in the ZA loop. However, this approach did not yield any improvement in enthalpy prediction and thus other factors must be at play here.

## Discussion

Although much work has been done on model systems^12,73,74^ the increase in the number of degrees of freedom associated with protein–ligand systems that are the size of typical drug-targets has led to an apparent reticence to explore enthalpy predictions and their potential utility. Given the growth in available computational power, we reasoned it would be useful to investigate the level of accuracy (and precision) one might obtain for a well-characterized protein–ligand system. To that end we focussed on bromodomains, a system for which we have previously shown that accurate Δ*G* predictions could be obtained *via* absolute binding free energies both for different compounds^15^ at the same protein and for the same compound at different proteins.^21^

We focussed on the BRD4-1 system as it is well characterized both in terms of structure and biophysical characterizations. We first asked the question of how accurate the calculation of enthalpy for this system would be assuming standard procedures reported in the literature, similar to our previous approach for Δ*G* predictions.^15^ For the AMBER forcefield, the results were surprisingly accurate and precise (Fig. 3) with only three obvious outliers from a 2 kcal mol^−1^ error boundary, thus suggesting that it is indeed possible to make reasonably accurate predictions of enthalpy. Such performance is remarkably encouraging. Outliers provide an opportunity to gain more insight into what factors are important in the prediction of enthalpy. Parameterization is certainly one aspect that can offer improvement as indeed was found for some systems here when charges were optimized. This aspect is also highlighted by the direct comparison between force-fields (Fig. 9a), where it is notable that AMBER tends to overpredict and both OPLS and CHARMM tend to underpredict Δ*H*. It is also the case the outliers predicted for AMBER are not the same as those for OPLS and CHARMM.

Comparison of the force-fields suggests that OPLS and AMBER were of comparable (similar RMSE), while CHARMM tended to give worse predictions, likely as a consequence of ligand parameters in CGenFF requiring further optimization. This is consistent with our previous observations on cucurbit[7]uril–guest systems where CGenFF performed worse compared to other force fields.^75^ The observations here reinforce the need for caution and care to be taken when using new parameters.

However, in some instances force-field was clearly not the major influence, and this led us ultimately to the identification of the influence of the ZA loop on the enthalpy prediction.

The dynamics and flexibility of the ZA loop has been reported across many different bromodomains, including for example ATAD2,^76,77^ BRD2-2,^78^ BRD4-1,^79,80^ BRPF1,^81^ BAZ2B,^82,83^ BRD9,^84^ BRG1 (ref. 85) and CBP.^81^ Thus, it has been proposed that the dynamic nature of the ZA-loop plays an important role in selectivity due to its flexibility and sequence variation between bromodomains.^17,82,86^ However, experimental 3D structures in the PDB databank mostly show no significant changes in the secondary structure despite the apparent plasticity. A recent work reported an investigation of 297 crystal structures of BRD4-1 and concluded that there is a high level of similarity in the binding pocket region, regardless of the bound ligand.^87^ Nevertheless, there are many studies highlighting flexibility. Eron *et al.*, using hydrogen–deuterium exchange mass spectrometry (HDX-MS), reported significant deuterium uptake on the ZA-loop of the apo state, but in contrast, the solvent shielding data pointed out a high degree of stabilization of the ZA-loop with the CFT-1297 degrader ligand.^88^ Yu *et al.* reported significant chemical shift around the ZA-loop for BRD4-1 upon ligand binding even though they showed that there were no significant differences between the solution structure of BRD4-1 and its crystal structure in the backbone resonance assignment data.^89^ Furthermore, ATAD2 displays “open” or “closed” conformational states of the ZA-loop.^76,90–92^ Further examples from BRG1,^85^ BRD7 (ref. 93) and BRD2-2,^78^ show large structural deviations in the ZA-loop when compared to the other regions according to NMR experiments.

Moreover, computational studies have provided detailed analysis of the dynamic nature of the ZA-loop.^26,31,94–96^ Tumdam, R., *et al.* showed that the ZA-loop in the apo-BRD4-1 can adopt a similar conformation to that which we observe here.^96^ The nature of the transitions of the ZA loop we observe appear very similar to that reported by Heinzelmann *et al.*^26^ who also computed that the free energy difference was 2.54 kcal mol^−1^ (with TIP3P) more favourable for their “open state” (equivalent to our ZA2 state here). Additionally, Cheng *et al.* showed an “in/out” transition of the ZA-loop in BRD4-1 using QM/MM, explaining the differential binding of RVX-208 & 297.^97^ More recently, a hidden state across all bromodomain families was proposed *via* used MD simulations and Markov state modelling in which important backbone hydrogen bonds are broken and the ZA-loop displaces away from the α_A_ helix.^29^

Seven of the ten complexes here gave excellent predictions of enthalpy of binding. For six of those complexes, most of the simulation time is spent in the ZA1 (close to crystal) conformation. The 5DW2 complex gives an excellent prediction of enthalpy, but rather interesting, readily transitions to the ZA2 loop conformation and indeed using only these conformations gives much more accurate predictions. Analysis of the 5DW2 simulations with the ZA2 conformations reveals that the ligand makes more close contacts (<0.4 A) with Asp88 and Tyr139 in ZA2 than ZA1 (Fig. S10†).

Of the three initial outliers with AMBER, simulations of the olinone (30M) complex (4QB3) gave some improvement when charges were refined with QM calculations. However, in these simulations, the complex occupied more time in the ZA1 conformation and it appears this conformation gives a more accurate estimate of free energy (with AMBER). Interestingly, olinone appears to make interactions with the BC loop in both conformations.

Accurate enthalpy predictions for bromosporine (BMF) in complex with BRD4-1 (5IGK) were only possible when combining predictions from both ZA1 and ZA2 conformations. Whilst simulations of the BzT-7 (08K) complex (3U5L) demonstrate that more accurate predictions can be obtained with just the ZA2 conformation, even though this conformation was never transitioned to in the initial set of 20 simulations. It may be the BzT-7 creates an energy barrier for the loop transition, though this is likely an indirect effect as BzT-7 is one of the smaller ligands and does not interact directly with the ZA loop. Further work would be necessary to explore this in more detail.

Of course, enthalpy is only one component of the binding free energy, Δ*G*. The results above demonstrate that the interaction and behaviour of a small, but crucial loop, near the binding site plays a key role in shaping that component. The calculations performed here were all retrospective, but such calculations in the future will only be useful if they can be useful prospectively (*i.e.* where we do not know/have the ITC measurements). What do these results mean in that context? Firstly, the trends in the initial data are reasonably good. Secondly, if one has confidence in the parameters, then sampling of key conformation states is a key issue (as would be expected). If one has knowledge of important (for ligand-binding) dynamics up-front, then strategies can be incorporated to mitigate this. Thirdly, if one has ITC data and Δ*G* calculations are in agreement but Δ*H* are not, that could be indicative of hidden states/cryptic sites and thus open up new avenues for design against existing targets. The Δ*G* would hide the presence of these states through entropy-enthalpy compensation. At the very least this would enable one to approach the predicted values with the necessary caution for sensible interpretations. Thus, in a prospective scenario, if one observes conformational transitions (in for example loops as here) and obtains enthalpy values that differ by several kcal mol^−1^, that should at least suggest that great care should be taken over any future interpretation of the thermodynamics.

## Conclusions

We have demonstrated that absolute ligand-binding enthalpy calculations for a well-characterized drug-target, BRD4-1, can give reasonably accurate results. Our results clearly show a strong dependence on the behaviour of the ZA loop to the predicted enthalpy for AMBER but this is less apparent in the OPLS simulations. We have also demonstrated that this alternative loop conformation is likely readily accessible, if not dominant, in the apo state in solution and that crystal lattice packing likely constrains the conformation. Indeed, it may well be the case that the ZA1 conformation of the loop is particularly amenable to lattice formation and thus the reason why many complexes exhibit this conformation. This observation highlights the need to take particular care when using apo state for docking studies and in the subsequent processes of rational drug design, like FEP calculations.

A key question that remains very open at this stage is just how generalizable is this approach in giving accurate predictions of Δ*H*. Can we expect this approach to become prospective? Prediction of Δ*H* may well be useful in the context of trying to optimize enthalpic contributions during a drug-discovery campaign. However, to do that with confidence in a prospective fashion will be dependent on more studies showing the approach can deliver across a variety of different systems. The work here should be taken as evidence and encouragement that it is feasible, at least for some systems. Studies on additional systems would also allow us to begin to understand how strong entropic contributions (by inference) might be linked to particular moieties or water molecules.

An alternative way to use this approach however, might be to draw researchers attention to hidden conformational states that from the initial structural biology work may not be immediately apparent. Outliers could be a way to identify such behaviour. The predicted contribution of different states to the enthalpic signature may provide a useful metric with which to gauge the importance of different states and how valuable they might be in terms of targeting. The ability to make accurate enthalpy predictions alongside accurate Δ*G* free energy predictions moves us considerably closer to being able to design ligands with desired thermodynamic properties, something that has long been sought after.^98^ Indeed, for some systems like membrane proteins these approaches will be very important as experimentally they are often more difficult to work with. This will be significantly easier if one has a good understanding of the dynamics of the protein before commencing such studies.

## Data availability

Input coordinate and parameters files used in this study have been deposited at: https://doi.org/10.5281/zenodo.7534582.

## Author contributions

PCB formulated the project. SSC performed all simulations. SSC and PCB analysed the data. SSC wrote the first draft and both authors contributed to revising and editing the manuscript.

## Conflicts of interest

There are no conflicts to declare.

## Supplementary Material

SC-014-D2SC06471E-s001

SC-014-D2SC06471E-s002

## References

[cit1] Chipot C. and Pohorille A., Free energy calculations: theory and applications in chemistry and biology, Springer, 2007

[cit2] Shirts M. R. , Mobley D. L. and Chodera J. D., in Annual Reports in Computational Chemistry, ed. D. C. Spellmeyer and R. Wheeler, Elsevier, 2007, ch. 4, vol. 3, pp. 41–59

[cit3] Mey A. S. J. S., Allen B. K., Bruce Macdonald H. E., Chodera J. D., Hahn D. F., Kuhn M., Michel J., Mobley D. L., Naden L. N., Prasad S., Rizzi A., Scheen J., Shirts M. R., Tresadern G., Xu H. (2021). Living J. Comput. Mol. Sci..

[cit4] Levy R. M., Gallicchio E. (1998). Annu. Rev. Phys. Chem..

[cit5] Lu N., Kofke D. A., Woolf T. B. (2003). J. Phys. Chem. B.

[cit6] Roy A., Hua D. P., Ward J. M., Post C. B. (2014). J. Chem. Theory Comput..

[cit7] Wyczalkowski M. A., Vitalis A., Pappu R. V. (2010). J. Phys. Chem. B.

[cit8] Freire E. (2008). Drug Discovery Today.

[cit9] Chodera J. D., Mobley D. L. (2013). Annu. Rev. Biophys..

[cit10] Fox J. M., Zhao M., Fink M. J., Kang K., Whitesides G. M. (2018). Annu. Rev. Biophys..

[cit11] Fenley A. T., Henriksen N. M., Muddana H. S., Gilson M. K. (2014). J. Chem. Theory Comput..

[cit12] Gao K., Yin J., Henriksen N. M., Fenley A. T., Gilson M. K. (2015). J. Chem. Theory Comput..

[cit13] Henriksen N. M., Fenley A. T., Gilson M. K. (2015). J. Chem. Theory Comput..

[cit14] Li A., Gilson M. K. (2018). J. Chem. Phys..

[cit15] Aldeghi M., Heifetz A., Bodkin M. J., Knapp S., Biggin P. C. (2016). Chem. Sci..

[cit16] Mobley D. L., Gilson M. K. (2017). Annu. Rev. Biophys..

[cit17] Filippakopoulos P., Knapp S. (2012). FEBS Lett..

[cit18] Shu S., Polyak K. (2016). Cold Spring Harbor Symp. Quant. Biol..

[cit19] Wang C.-Y., Filippakopoulos P. (2015). Trends Biochem. Sci..

[cit20] Aldeghi M., Bodkin M. J., Knapp S., Biggin P. C. (2017). J. Chem. Inf. Model..

[cit21] Aldeghi M., Heifetz A., Bodkin M. J., Knapp S., Biggin P. C. (2017). J. Am. Chem. Soc..

[cit22] Aldeghi M., Ross G. A., Bodkin M. J., Essex J. W., Knapp S., Biggin P. C. (2018). Commun. Chem..

[cit23] Brand M., Clayton J., Moroglu M., Schiedel M., Picaud S., Bluck J. P., Skwarska A., Bolland H., Chan A. K. N., Laurin C. M. C., Scorah A. R., See L., Rooney T. P. C., Andrews K. H., Fedorov O., Perell G., Kalra P., Vinh K. B., Cortopassi W. A., Heitel P., Christensen K. E., Cooper R. I., Paton R. S., Pomerantz W. C. K., Biggin P. C., Hammond E. M., Filippakopoulos P., Conway S. J. (2021). J. Med. Chem..

[cit24] Dickson A. (2018). Biophys. J..

[cit25] Guest E. E., Cervantes L. F., Pickett S. D., Brooks C. L., Hirst J. D. (2022). J. Chem. Inf. Model..

[cit26] Heinzelmann G., Henriksen N. M., Gilson M. K. (2017). J. Chem. Theory Comput..

[cit27] Jennings L. E., Schiedel M., Hewings D. S., Picaud S., Laurin C. M. C., Bruno P. A., Bluck J. P., Scorah A. R., See L., Reynolds J. K., Moroglu M., Mistry I. N., Hicks A., Guzanov P., Clayton J., Evans C., Stazi G., Biggin P. C., Mapp A. K., Hammond E. M., Humphreys P. G., Filippakopoulos P., Conway S. J. (2018). Bioorg. Med. Chem..

[cit28] Laurin C. M. C., Bluck J. P., Chan A. K. N., Keller M., Boczek A., Scorah A. R., See K. F. L., Jennings L. E., Hewings D. S., Woodhouse F., Reynolds J. K., Schiedel M., Humphreys P. G., Biggin P. C., Conway S. J. (2021). ACS Infect. Dis..

[cit29] Raich L. , Meier K., Günther J., Christ C. D., Noe F. and Olsson S., bioRxiv, 2020, preprint, 10.1101/2020.04.01.019547

[cit30] Wan S., Bhati A. P., Zasada S. J., Wall I., Green D., Bamborough P., Coveney P. V. (2017). J. Chem. Theory Comput..

[cit31] Zhang X., Chen K., Wu Y.-D., Wiest O. (2017). PLoS One.

[cit32] Backman T. W. H., Cao Y., Girke T. (2011). Nucleic Acids Res..

[cit33] Cao Y., Charisi A., Cheng L.-C., Jiang T., Girke T. (2008). Bioinformatics.

[cit34] Pettersen E. F., Goddard T. D., Huang C. C., Couch G. S., Greenblatt D. M., Meng E. C., Ferrin T. E. (2004). J. Comput. Chem..

[cit35] Jo S., Cheng X., Lee J., Kim S., Park S.-J., Patel D. S., Beaven A. H., Lee K. I., Rui H., Park S., Lee H. S., Roux B., MacKerell Jr A. D., Klauda J. B., Qi Y., Im W. (2017). J. Comput. Chem..

[cit36] Jo S., Kim T., Iyer V. G., Im W. (2008). J. Comput. Chem..

[cit37] He X., Man V. H., Yang W., Lee T.-S., Wang J. (2020). J. Chem. Phys..

[cit38] Jakalian A., Jack D. B., Bayly C. I. (2002). J. Comput. Chem..

[cit39] Jakalian A., Bush B. L., Jack D. B., Bayly C. I. (2000). J. Comput. Chem..

[cit40] Maier J. A., Martinez C., Kasavajhala K., Wickstrom L., Hauser K. E., Simmerling C. (2015). J. Chem. Theory Comput..

[cit41] Mark P., Nilsson L. (2001). J. Phys. Chem. A.

[cit42] Huang J., Rauscher S., Nawrocki G., Ran T., Feig M., de Groot B. L., Grubmueller H., MacKerell Jr A. D. (2017). Nat. Methods.

[cit43] Robertson M. J., Tirado-Rives J., Jorgensen W. L. (2015). J. Chem. Theory Comput..

[cit44] Vanommeslaeghe K., MacKerell Jr A. D. (2012). J. Chem. Inf. Model..

[cit45] Vanommeslaeghe K., Raman E. P., MacKerell Jr A. D. (2012). J. Chem. Inf. Model..

[cit46] Vanommeslaeghe K., Hatcher E., Acharya C., Kundu S., Zhong S., Shim J., Darian E., Guvench O., Lopes P., Vorobyov I., MacKerell Jr A. D. (2010). J. Comput. Chem..

[cit47] Dodda L. S., de Vaca I. C., Tirado-Rives J., Jorgensen W. L. (2017). Nucleic Acids Res..

[cit48] Dodda L. S., Vilseck J. Z., Tirado-Rives J., Jorgensen W. L. (2017). J. Phys. Chem. B.

[cit49] Parrish R. M., Burns L. A., Smith D. G. A., Simmonett A. C., DePrince A. E., Hohenstein E. G., Bozkaya U., Sokolov A. Y., Di Remigio R., Richard R. M., Gonthier J. F., James A. M., McAlexander H. R., Kumar A., Saitow M., Wang X., Pritchard B. P., Verma P., Schaefer H. F., Patkowski K., King R. A., Valeev E. F., Evangelista F. A., Turney J. M., Crawford T. D., Sherrill C. D. (2017). J. Chem. Theory Comput..

[cit50] Galvelis R., Doerr S., Damas J. M., Harvey M. J., De Fabritiis G. (2019). J. Chem. Inf. Model..

[cit51] Páll S., Zhmurov A., Bauer P., Abraham M., Lundborg M., Gray A., Hess B., Lindahl E. (2020). J. Chem. Phys..

[cit52] Abraham M. J., Murtola T., Schulz R., Páll S., Smith J. C., Hess B., Lindahl E. (2015). SoftwareX.

[cit53] Berendsen H. J. C., van der Spoel D., van Drunen R. (1995). Comput. Phys. Commun..

[cit54] van der Spoel D., Lindahl E., Hess B., Groenhof G., Mark A. E., Berendsen H. J. C. (2005). J. Comput. Chem..

[cit55] Flyvbjerg H., Petersen H. G. (1989). J. Chem. Phys..

[cit56] Huang J., Rauscher S., Nawrocki G., Ran T., Feig M., de Groot B. L., Grubmüller H., MacKerell A. D. (2017). Nat. Methods.

[cit57] Robertson M. J., Tirado-Rives J., Jorgensen W. L. (2015). J. Chem. Theory Comput..

[cit58] Wickham H. , *ggplot2: Elegant Graphics for Data Analysis*, Springer-Verlag, New York, 2009

[cit59] Hügle M., Regenass P., Warstat R., Hau M., Schmidtkunz K., Lucas X., Wohlwend D., Einsle O., Jung M., Breit B., Günther S. (2020). J. Med. Chem..

[cit60] Zhang M., Zhang Y., Song M., Xue X., Wang J., Wang C., Zhang C., Li C., Xiang Q., Zou L., Wu X., Wu C., Dong B., Xue W., Zhou Y., Chen H., Wu D., Ding K., Xu Y. (2018). J. Med. Chem..

[cit61] Filippakopoulos P., Qi J., Picaud S., Shen Y., Smith W. B., Fedorov O., Morse E. M., Keates T., Hickman T. T., Felletar I., Philpott M., Munro S., McKeown M. R., Wang Y., Christie A. L., West N., Cameron M. J., Schwartz B., Heightman T. D., La Thangue N., French C. A., Wiest O., Kung A. L., Knapp S., Bradner J. E. (2010). Nature.

[cit62] Lucas X., Wohlwend D., Hügle M., Schmidtkunz K., Gerhardt S., Schüle R., Jung M., Einsle O., Günther S. (2013). Angew. Chem., Int. Ed..

[cit63] Gacias M., Gerona-Navarro G., Plotnikov A. N., Zhang G., Zeng L., Kaur J., Moy G., Rusinova E., Rodriguez Y., Matikainen B., Vincek A., Joshua J., Casaccia P., Zhou M.-M. (2014). Chem. Biol..

[cit64] Picaud S., Strocchia M., Terracciano S., Lauro G., Mendez J., Daniels D. L., Riccio R., Bifulco G., Bruno I., Filippakopoulos P. (2015). J. Med. Chem..

[cit65] Xue X., Zhang Y., Liu Z., Song M., Xing Y., Xiang Q., Wang Z., Tu Z., Zhou Y., Ding K., Xu Y. (2016). J. Med. Chem..

[cit66] Hügle M., Lucas X., Weitzel G., Ostrovskyi D., Breit B., Gerhardt S., Einsle O., Günther S., Wohlwend D. (2016). J. Med. Chem..

[cit67] Raux B., Voitovich Y., Derviaux C., Lugari A., Rebuffet E., Milhas S., Priet S., Roux T., Trinquet E., Guillemot J.-C., Knapp S., Brunel J.-M., Fedorov A. Y., Collette Y., Roche P., Betzi S., Combes S., Morelli X. (2016). J. Med. Chem..

[cit68] Montenegro R. C., Clark P. G. K., Howarth A., Wan X., Ceroni A., Siejka P., Nunez-Alonso G. A., Monteiro O., Rogers C., Gamble V., Burbano R., Brennan P. E., Tallant C., Ebner D., Fedorov O., Neill E., Knapp S., Dixon D., Müller S. (2016). Oncotarget.

[cit69] Picaud S., Leonards K., Lambert J.-P., Dovey O., Wells C., Fedorov O., Monteiro O., Fujisawa T., Wang C.-Y., Lingard H., Tallant C., Nikbin N., Guetzoyan L., Ingham R., Ley S. V., Brennan P., Muller S., Samsonova A., Gingras A.-C., Schwaller J., Vassiliou G., Knapp S., Filippakopoulos P. (2016). Sci. Adv..

[cit70] Fiala T., Sleziakova K., Marsalek K., Salvadori K., Sindelar V. (2018). J. Org. Chem..

[cit71] Le V. H., Yanney M., McGuire M., Sygula A., Lewis E. A. (2014). J. Phys. Chem. B.

[cit72] Bertin G. , https://hdl.handle.net/20.500.12608/21280, Padua, 2020

[cit73] Çınaroğlu S. S., Biggin P. C. (2021). J. Phys. Chem. B.

[cit74] Schönbeck C., Holm R. (2019). J. Phys. Chem. B.

[cit75] Cinaroglu S. S., Biggin P. C. (2021). J. Phys. Chem. B.

[cit76] Poncet-Montange G., Zhan Y., Bardenhagen J. P., Petrocchi A., Leo E., Shi X., Lee IV G. R., Leonard P. G., Geck Do M. K., Cardozo M. G., Andersen J. N., Palmer W. S., Jones P., Ladbury J. E. (2015). Biochem. J..

[cit77] Zhou Y., Hussain M., Kuang G., Zhang J., Tu Y. (2018). Physical Chemistry Chemical Physics.

[cit78] Huang H., Zhang J., Shen W., Wang X., Wu J., Wu J., Shi Y. (2007). BMC Struct. Biol..

[cit79] Cheng C., Diao H., Zhang F., Wang Y., Wang K., Wu R. (2017). PCCP Phys. Chem. Chem. Phys..

[cit80] Eron S. J., Huang H., Agafonov R. V., Fitzgerald M. E., Patel J., Michael R. E., Lee T. D., Hart A. A., Shaulsky J., Nasveschuk C. G., Phillips A. J., Fisher S. L., Good A. (2021). ACS Chem. Biol..

[cit81] Zhu J., Zhou C., Caflisch A. (2018). Eur. J. Med. Chem..

[cit82] Steiner S., Magno A., Huang D., Caflisch A. (2013). FEBS Lett..

[cit83] Ferguson F. M., Dias D. M., Rodrigues J. P. G. L. M., Wienk H., Boelens R., Bonvin A. M. J. J., Abell C., Ciulli A. (2014). Biochemistry.

[cit84] Theodoulou N. H., Bamborough P., Bannister A. J., Becher I., Bit R. A., Che K. H., Chung C.-w., Dittmann A., Drewes G., Drewry D. H., Gordon L., Grandi P., Leveridge M., Lindon M., Michon A.-M., Molnar J., Robson S. C., Tomkinson N. C. O., Kouzarides T., Prinjha R. K., Humphreys P. G. (2016). J. Med. Chem..

[cit85] Shen W., Xu C., Huang W., Zhang J., Carlson J. E., Tu X., Wu J., Shi Y. (2007). Biochemistry.

[cit86] Vidler L. R., Brown N., Knapp S., Hoelder S. (2012). J. Med. Chem..

[cit87] Guest E. E., Pickett S. D., Hirst J. D. (2021). Org. Biomol. Chem..

[cit88] Eron S. J., Huang H., Agafonov R. V., Fitzgerald M. E., Patel J., Michael R. E., Lee T. D., Hart A. A., Shaulsky J., Nasveschuk C. G., Phillips A. J., Fisher S. L., Good A. (2021). ACS Chem. Biol..

[cit89] Yu J.-l., Chen T.-t., Zhou C., Lian F.-l., Tang X.-l., Wen Y., Shen J.-k., Xu Y.-c., Xiong B., Zhang N.-x. (2016). Acta Pharmacol. Sin..

[cit90] Langini C., Caflisch A., Vitalis A. (2017). J. Biol. Chem..

[cit91] Lloyd J. T., McLaughlin K., Lubula M. Y., Gay J. C., Dest A., Gao C., Phillips M., Tonelli M., Cornilescu G., Marunde M. R., Evans C. M., Boyson S. P., Carlson S., Keogh M.-C., Markley J. L., Frietze S., Glass K. C. (2020). J. Med. Chem..

[cit92] Zhou Y., Hussain M., Kuang G., Zhang J., Tu Y. (2018). PCCP Phys. Chem. Chem. Phys..

[cit93] Sun H., Liu J., Zhang J., Shen W., Huang H., Xu C., Dai H., Wu J., Shi Y. (2007). Biochem. Biophys. Res. Commun..

[cit94] Kuang M., Zhou J., Wang L., Liu Z., Guo J., Wu R. (2015). J. Chem. Inf. Model..

[cit95] Su J., Liu X., Zhang S., Yan F., Zhang Q., Chen J. (2018). J. Biomol. Struct. Dyn..

[cit96] Tumdam R., Kumar A., Subbarao N., Balaji B. S. (2018). SAR QSAR Environ. Res..

[cit97] Cheng C., Diao H., Zhang F., Wang Y., Wang K., Wu R. (2017). Phys. Chem. Chem. Phys..

[cit98] Klebe G. (2019). Drug Discovery Today.

