# Supplementary material for: The role of loop dynamics in the prediction of ligand–protein binding enthalpy
Source: Chem Sci. 2023 Jun 1;14(24):6792–805. doi: 10.1039/d2sc06471e (PMC10284145; doi:10.1039/d2sc06471e)
Supplement: SC-014-D2SC06471E-s001 [file SC-014-D2SC06471E-s001.pdf]

Electronic Supplementary Material (ESI) for Chemical Science.
This journal is © The Royal Society of Chemistry 2023

Supporting Information

The Role of Loop Dynamics in the Prediction of Ligand-Protein Binding Enthalpy

Süleyman Selim Çınaroğlu and Philip C. Biggin*

Supporting Information

Table of Contents

TABLES **3**

Table S1: Initial BRD4-1 set having PDB structures with ITC binding data *3*
Table S2: Initial binding enthalpy calculations *4*
Table S3: Binding enthalpy calculations for 3UL5, 4BQ3 and 5IGK with optimized charges, differing buffer conditions and ZA-loop conformations *5*
Table S4: Binding enthalpy calculations from specific ZA loop conformations *6*
Table S5. Percentage occupation of ZA loop states *7*
Table S6. Physical determinants of Binding Enthalpies *8*
Table S7. Binding enthalpies calculated by using three different force fields. *9*
Table S8: Number of penalties (higher than 50) obtained from the CGenFF server. *10*
Table S9. Absolute differences between calculated binding enthalpies and experimental values for the 3U5L and 5IGK systems. *11*

FIGURES **12**

Figure S1. Enthalpy-Entropy Compensation in BRD4-1 calorimetry data. *12*
Figure S2. The change in the average potential energy of cumulative simulation data from 20 replicates for all complexes along with the respective blocking analysis curves. *13*
Figure S3. Effects of parameters and buffer. *14*
Figure S4. Three different ionization states of HEPES buffer. *15*
Figure S5. Violin plots for all-atom RMSD of ligands with respect to their starting conformations. *16*
Figure S6. Pairwise RMSD for ZA-loop (76-106 residues) backbone atoms of the simulation in Figure 6A of the main manuscript. *17*
Figure S7. RMSD violin plots for ZA-loop (76-106 residues) backbone atoms of 3U5L and 5IGK for simulations having ZA1 (blue) and ZA2 (purple) in the starting structure. *18*
Figure S8. A) The ω (CA-C-N-CA) angle distribution of Gln84 (in the ZA loop) across 100 apo-receptor simulations. *19*
Figure S9. RMSD violin plots for the ZA-loop (76-106 residues) backbone atoms in crystal lattice simulations. *20*
Figure S10. Difference (ZA1 – ZA2) of the average number of contacts between conformations. *21*
Figure S11. RMSD violin plots for ZA-loop (76-106 residues -see Fig. 1.) backbone atoms of 2OSS apo-receptor and complex simulations after fitting whole protein backbone. *22*
Figure S12. The overall sampling distribution of the simulations by dihedral angle distributions. *23*
Figure S13. Convergence pattern of the calculated ΔH. *24*
Figure S14: OPLS introduced greater flexibility to the ZA loop of 5D3S. *25*

REFERENCES **26**

TABLES

Table S1: Initial BRD4-1 set having PDB structures with ITC binding data

PDB ID	Cluster Size	Cluster ID	K_d (M)	∆G (kcal/mol)	ITC Condition	Ref.
3MXF	1	2	4.9E-08	-9.64	50 mM HEPES, 150 mM NaCl, and pH 7.4	1
3P5O	3	3	9.5E-08	-9.59	20 mM HEPES, 100 mM NaCl, and pH 7.5	2
3U5J	3	3	0.00000246	-7.40	50 mM HEPES, 150 mM NaCl, and pH 7.4	3
3U5L	3	3	0.00000064	-8.16	50 mM HEPES, 150 mM NaCl, and pH 7.4	3
4E96	6	6	4.74E-08	-9.68	50 mM HEPES, 100 mM NaCl, 0.5 mM TCEP and pH 7.5	4
4J3I	2	7	1.142E-06	-7.84	50 mM HEPES, 150 mM NaCl, 0.05% DMSO and pH 7.5	5
4MR4	2	7	0.00000893	-6.87	50 mM HEPES, 150 mM NaCl, and pH 7.5	6
4LYS	2	8	0.000046	-5.90	Not available	7
4LYW	11	9	2.37E-07	-9.00	Not available	7
4LZR	2	8	0.00002	-6.40	Not available	7
4LZS	11	9	0.000016	-6.60	Not available	7
4OGI	1	12	3.7E-08	-9.79	50 mM HEPES, 150 mM NaCl, and pH 7.4	8
4OGJ	1	13	1.64E-07	-8.94	50 mM HEPES, 150 mM NaCl, and pH 7.4	8
4QB3	1	14	0.0000033	-7.55	50 mM Na_3PO_4, 150 mM NaCl, and pH 7.4	9
4XY9	2	15	4.651E-06	-7.03	50 mM HEPES, 150 mM NaCl, and pH 7.5	10
4XYA	2	15	0.00000137	-7.73	50 mM HEPES, 150 mM NaCl, and pH 7.5	10
5BT4	1	17	8.85E-07	-8.14	50 mM HEPES, 150 mM NaCl, and pH 7.5	11
5CP5	6	6	1.234E-06	-8.06	50 mM HEPES, 150 mM NaCl, 0.5 mM TCEP and pH 7.5	12
5CQT	6	6	1.24E-07	-9.42	50 mM HEPES, 150 mM NaCl, 0.5 mM TCEP and pH 7.5	12
5CY9	6	6	2.18E-07	-9.09	50 mM HEPES, 150 mM NaCl, 0.5 mM TCEP and pH 7.5	12
5D0C	6	6	0.00000234	-7.68	50 mM HEPES, 150 mM NaCl, 0.5 mM TCEP and pH 7.5	12
5D24	11	9	5.841E-06	-7.14	Not available	13
5D25	11	9	5.075E-07	-8.59	Not available	13
5D26	11	9	8.096E-07	-8.31	Not available	13
5D3H	11	9	0.00000793	-6.96	Not available	13
5D3J	11	9	5.733E-06	-7.15	Not available	13
5D3L	11	9	8.844E-07	-8.26	Not available	13
5D3N	11	9	9.202E-06	-6.87	Not available	13
5D3P	11	9	7.391E-06	-7.00	Not available	13
5D3S	11	9	1.272E-06	-8.04	Not available	13
5D3T	11	9	0.00001206	-6.71	Not available	13
5DW2	2	7	6.95E-07	-8.00	50 mM HEPES, 150 mM NaCl, and pH 7.5	14
5DX4	6	6	1.37E-07	-9.36	50 mM HEPES, 150 mM NaCl, 0.5 mM TCEP and pH 7.5	12
5EGU	1	1	0.0000014	-8.20	10 mM HEPES, 150 mM NaCl, and pH 7.5	15
5FBX	1	33	5.43E-09	-10.90	20 mM HEPES, 150 mM NaCl, 0.5 mM TCEP and pH 7.5	16
5IGK	1	34	4.18E-08	-9.73	50 mM HEPES, 150 mM NaCl, and pH 7.5	17

Table S2: Initial binding enthalpy calculations

PDB	$E_{Complex}$	E_{Water}	$E_{Receptor}$	E_{Ligand}	ΔH_{MD}	$\Delta H_{Exp.}$
3MXF	-194,542.80 ± 0.47	-19,123.03 ± 0.07	-194,552.58 ± 0.24	-19,104.10 ± 0.09	-9.15 ± 0.54	-8.42
3U5L	-194,517.73 ± 0.40	-19,123.03 ± 0.07	-194,552.58 ± 0.24	-19,077.60 ± 0.07	-10.57 ± 0.47	-6.16
4LZR	-194,515.99 ± 0.64	-19,123.03 ± 0.07	-194,552.58 ± 0.24	-19,076.84 ± 0.07	-9.60 ± 0.69	-9.00
4QB3	-194,625.18 ± 0.71	-19,123.03 ± 0.07	-194,552.58 ± 0.24	-19,185.19 ± 0.07	-10.43 ± 0.75	-6.62
4XY9	-194,805.96 ± 1.07	-19,123.03 ± 0.07	-194,552.58 ± 0.24	-19,369.69 ± 0.07	-6.71 ± 1.10	-6.09
5D0C	-194,662.30 ± 0.64	-19,123.03 ± 0.07	-194,552.58 ± 0.24	-19,220.96 ± 0.07	-11.78 ± 0.69	-10.20
5D3S	-194,622.99 ± 0.88	-19,123.03 ± 0.07	-194,552.58 ± 0.24	-19,184.16 ± 0.07	-9.28 ± 0.92	-9.77
5DW2	-194,670.10 ± 0.58	-19,123.03 ± 0.07	-194,552.58 ± 0.24	-19,231.58 ± 0.07	-8.96 ± 0.63	-10.10
5FBX	-194,559.37 ± 1.09	-19,123.03 ± 0.07	-194,552.58 ± 0.24	-19,113.12 ± 0.09	-16.69 ± 1.13	-15.57
5IGK	-194,782.42 ± 0.58	-19,123.03 ± 0.07	-194,552.58 ± 0.24	-19,337.15 ± 0.07	-15.71 ± 0.64	-11.09

All values are in kcal/mol. Outliers are highlighted in bold. The error in ΔH_{MD} is taken as the maximum value of the error from the blocking analysis (Figure S2).

Table S3: Binding enthalpy calculations for 3UL5, 4BQ3 and 5IGK with optimized charges, differing buffer conditions and ZA-loop conformations.

PDB	$E_{Complex}$	E_{Water}	$E_{Receptor}$	E_{Ligand}	ΔH_{MD}	$\Delta H_{Exp.}$
3U5L[a]	-194,482.19 ± 0.41	-19,123.03 ± 0.07	-194,552.58 ± 0.24	-19,038.78 ± 0.07	-13.85 ± 0.48	-6.16
4QB3[a]	-194,507.71 ± 0.68	-19,123.03 ± 0.07	-194,552.58 ± 0.24	-19,073.03 ± 0.07	-5.13 ± 0.72	-6.62
5IGK[a]	-194,492.98 ± 0.53	-19,123.03 ± 0.07	-194,552.58 ± 0.24	-19,049.07 ± 0.07	-14.35 ± 0.59	-11.09
3U5L[b]	-202,904.22 ± 1.82	-19,123.03 ± 0.07	-202,979.60 ± 1.85	-19,038.78 ± 0.07	-8.86 ± 2.59	-6.16
5IGK[b]	-202,920.04 ± 2.97	-19,123.03 ± 0.07	-202,979.60 ± 1.85	-19,049.07 ± 0.07	-14.39 ± 3.50	-11.09
3U5L[c]	-194,474.86 ± 0.58	-19,123.03 ± 0.07	-194,552.58 ± 0.24	-19,038.78 ± 0.07	-6.52 ± 0.64	-6.16
3U5L[d]	-194,479.27 ± 3.21	-19,123.03 ± 0.07	-194,552.58 ± 0.24	-19,038.78 ± 0.07	-10.93 ± 3.22	-6.16
5IGK[c]	-194,485.25 ± 0.60	-19,123.03 ± 0.07	-194,552.58 ± 0.24	-19,049.07 ± 0.07	-6.62 ± 0.66	-11.09
5IGK[d]	-194,489.92 ± 3.06	-19,123.03 ± 0.07	-194,552.58 ± 0.24	-19,049.07 ± 0.07	-11.29 ± 3.07	-11.09

[a] Calculated by using simulations with optimized ligand parameters of 3UL5, 4BQ3, 5IGK
[b] Calculated by simulations with three ionization states of HEPES and NaCl
[c] Simulations were started using receptor with ZA2 conformation for complex simulations
[d] Calculated by using combined simulations data for both ZA1 and ZA2 from 40 simulations
All values are in kcal/mol.

Table S4: Binding enthalpy calculations from specific ZA loop conformations

PDB	$E_{Complex}$	ΔH_{MD}	$\Delta H_{Exp.}$
4LZR_ZA1	-194517.48 ± 0.22	-10.96 ± 0.25	-9.00
4QB3_ZA1	-194507.94 ± 0.20	-5.23 ± 0.23	-6.62
4XY9_ZA1	-194806.62 ± 0.23	-7.25 ± 0.25	-6.09
5DW2_ZA1	-194668.91 ± 0.23	-7.66 ± 0.25	-10.10
4LZR_ZA2	-194513.13 ± 0.32	-6.61 ± 0.34	-9.00
4QB3_ZA2	-194505.80 ± 0.45	-3.09 ± 0.46	-6.62
4XY9_ZA2	-194803.31 ± 0.31	-3.93 ± 0.33	-6.09
5DW2_ZA2	-194671.73 ± 0.31	-10.48 ± 0.33	-10.10

All values are in kcal/mol.

Table S5. Percentage occupation of ZA loop states

System	ZA1 (%)	ZA2 (%)	Intermediate (%)
3MXF	92.70	3.02	4.28
3U5L	99.69	0.00	0.31
3U5L_ion	91.70	6.20	2.10
3U5L_qm	99.33	0.00	0.67
3U5L_ZA	0.00	93.22	6.78
4LZR	64.75	26.94	8.32
4QB3	66.79	28.70	4.51
4QB3_qm	80.89	15.95	3.16
4XY9	65.03	31.49	3.48
5D0C	99.79	0.00	0.21
5D3S	99.97	0.00	0.03
5DW2	58.97	31.98	9.06
5FBX	99.88	0.00	0.12
5IGK	99.97	0.00	0.03
5IGK_ion	99.97	0.00	0.03
5IGK_qm	99.96	0.00	0.04
5IGK_ZA	0.66	88.27	11.06
receptor(20)[a]	19.74	74.53	5.73
receptor(100)[b]	20.98	73.27	5.76
receptor_ion	49.38	42.21	8.41

_ion; contains 50 mM HEPES and 150 mM NaCl, and also optimized ligand parameters
_qm; performed using optimized ligand parameters
_ZA: ZA2 was used as a starting structure
[a] Percentages for the first 20 repeats
[b] Percentages for all 100 repeats

ZA1 state is defined as: If the Val90:H-Val87:O and Lys91:H-Val87:O hydrogen bond distances are more than 4 Å and 5 Å respectively. ZA2 state is defined as: if the Val90:H-Val87:O and Lys91:H-Val87:O hydrogen bond distances are both less than 3 Å. Everything else is defined as intermediate.

Table S6. Physical determinants of Binding Enthalpies

PDB	Val	Coul	LJ
3MXF	0.38	-5.09	-4.35
3U5L_ZA1	1.63	-12.10	-3.21
3U5L_ZA2	-1.10	0.64	-5.90
4LZR	-1.04	-7.25	-1.31
4QB3	4.95	-6.70	-3.04
4XY9	-0.94	-2.13	-3.04
5D0C	1.68	-9.24	-3.78
5D3S	1.77	-5.81	-4.99
5DW2	0.90	-6.10	-3.28
5FBX	-0.94	-11.85	-3.90
5IGK_ZA1	3.53	-16.00	-1.75
5IGK_ZA2	1.06	-2.20	-5.37

Coul: Coulombic electrostatic contribution. **Val**: contribution from changes in bond-stretch, angle-bend, and dihedral terms. **LJ**: Lennard-Jones contribution.

Table S7. Binding enthalpies calculated by using three different force fields.

PDB	Exp.	AMBER	CHARMM	OPLS
3MXF	-8.42	-10.18 ± 0.92	-2.93 ± 1.24	-9.06 ± 0.93
3U5L	-6.16	-11.61 ± 0.88	-3.02 ± 1.58	-7.83 ± 1.05
4LZR	-9.00	-10.63 ± 1.01	-3.54 ± 1.35	-2.28 ± 1.15
4QB3	-6.62	-11.47 ± 1.06	-6.13 ± 1.36	-5.75 ± 1.06
4XY9	**-6.09**	**-7.75 ± 1.33**	**-7.00 ± 1.68**	**-7.39 ± 1.41**
5D0C	-10.20	-12.82 ± 1.01	12.11 ± 1.21	-12.35 ± 1.05
5D3S	-9.77	-10.31 ± 1.18	-3.53 ± 1.38	-5.42 ± 1.26
5DW2	-10.10	-10.00 ± 0.98	-5.47 ± 1.21	-8.91 ± 1.12
5FBX	-15.57	-17.73 ± 1.35	-10.82 ± 1.69	-10.17 ± 0.95
5IGK	-11.09	-16.75 ± 0.98	-10.97 ± 1.22	-10.73 ± 1.30

Binding enthalpies using AMBER are calculated without considering ZA-loop and ligand parameter optimization. All values are in kcal/mol. Bold entry gives binding enthalpies within the 2 kcal/mol error limit for all cases.

Table S8: Number of penalties (higher than 50) obtained from the CGenFF server.

PDB	Partial Charge	Parameter
3MXF[β]	3	22
3U5L	4	23
4LZR	8	25
4QB3[α]	0	0
4XY9[α]	3	3
5D0C[β]	0	3
5D3S[β]	0	1
5DW2[β]	0	2
5FBX	4	22
5IGK[α]	2	12

[α] Provided accurate binding enthalpies.

[β] Ligand left the binding pocket in at least one simulation.

Table S9. Absolute differences between calculated binding enthalpies and experimental values for the 3U5L and 5IGK systems.

Simulation	AMBER	OPLS	CHARMM
3U5L_ZA1	8.73	1.67	3.14
5IGK_ZA1	4.29	0.36	0.12
3U5L_ZA2	1.39	0.85	2.03
5IGK_ZA2	3.43	4.67	3.75
3U5L_All	5.81	0.48	0.72
5IGK_All	1.24	2.30	1.73

_ZA1: PDB structure was used as a starting structure.
_ZA2: ZA2 was used as a starting structure.
_All: Calculated by using combined simulations data for both ZA1 and ZA2 from 40 simulations

FIGURES

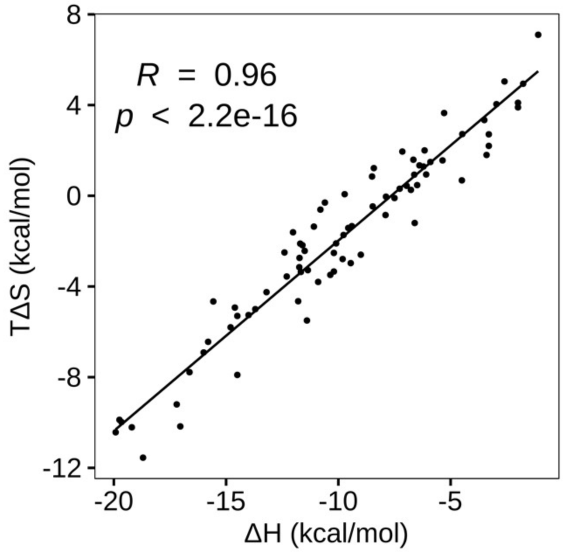

Figure S1. Enthalpy-Entropy Compensation in BRD4-1 calorimetry data.
The plot indicates a clear correlation between ΔH and TΔS components of the binding free energy.

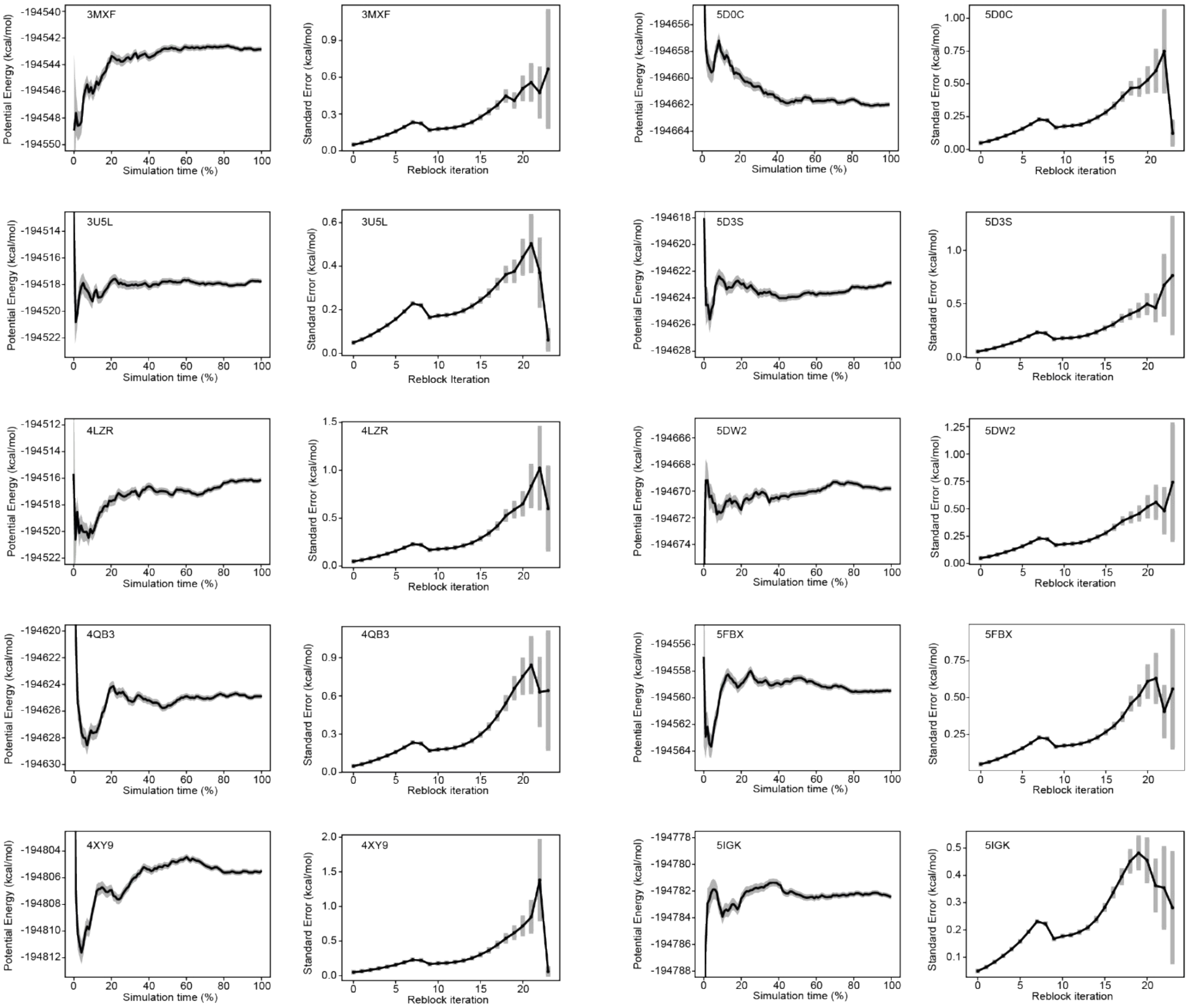

Figure S2. The change in the average potential energy of cumulative simulation data from 20 replicates for all complexes along with the respective blocking analysis curves.
Grey shade represents the standard error of the mean.

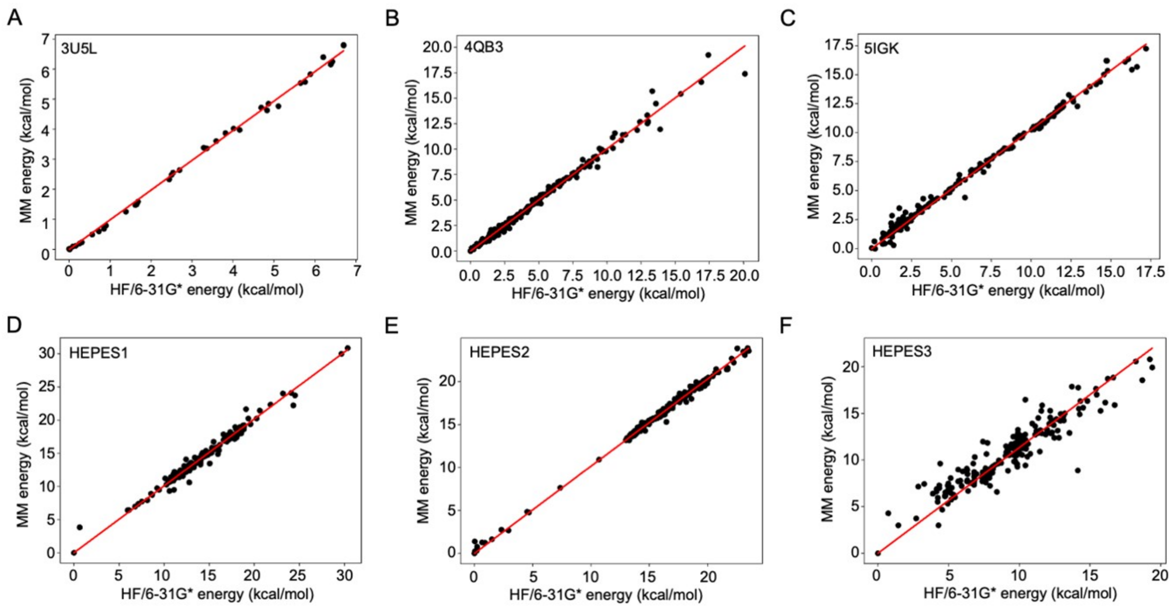

Figure S3. Effects of parameters and buffer**.**
(A-C) Comparison of HF/6-31G* and MM energies for 3UL5, 4BQ3, 5IGK derived from the parameterize software tool (https://software.acellera.com/docs/latest/parameterize [18] and also (D-F) three ionization states of HEPES (see Figure S4). Points represent different conformations and the energy as computed by the two methods.

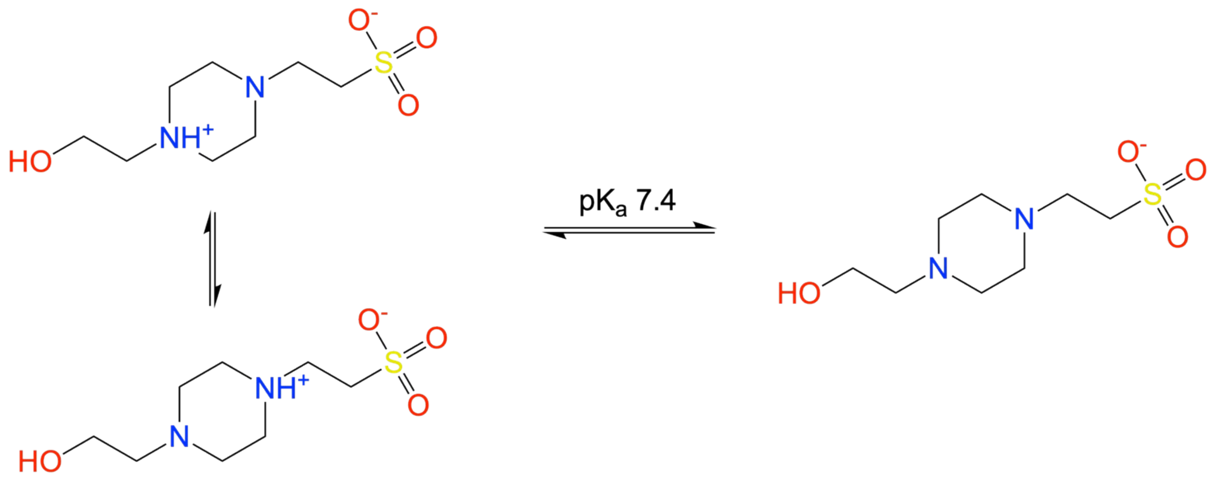

Figure S4. Three different ionization states of HEPES buffer.

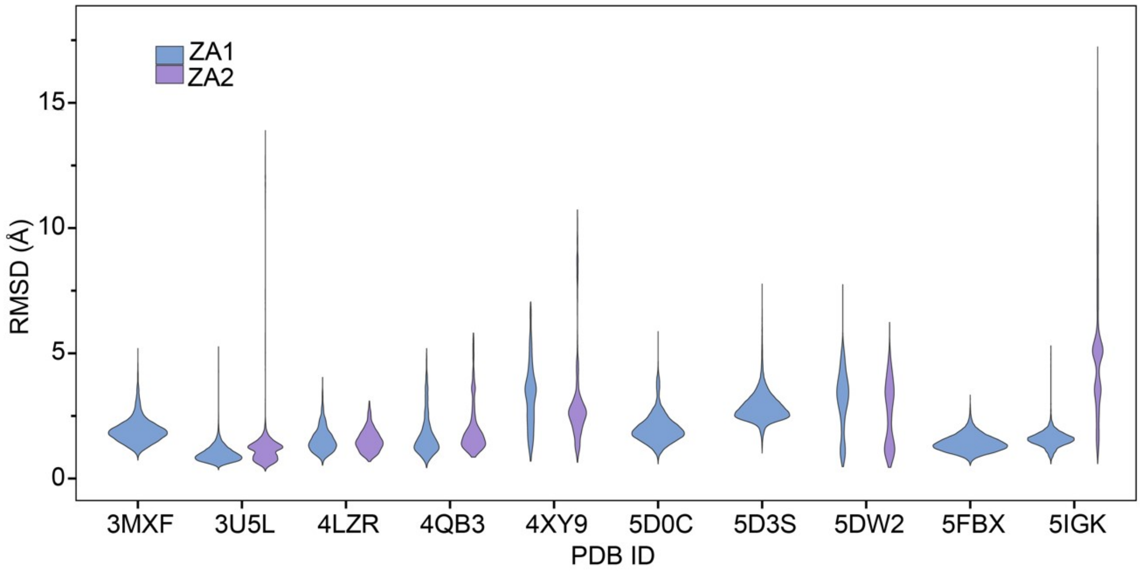

Figure S5. Violin plots for all-atom RMSD of ligands with respect to their starting conformations.

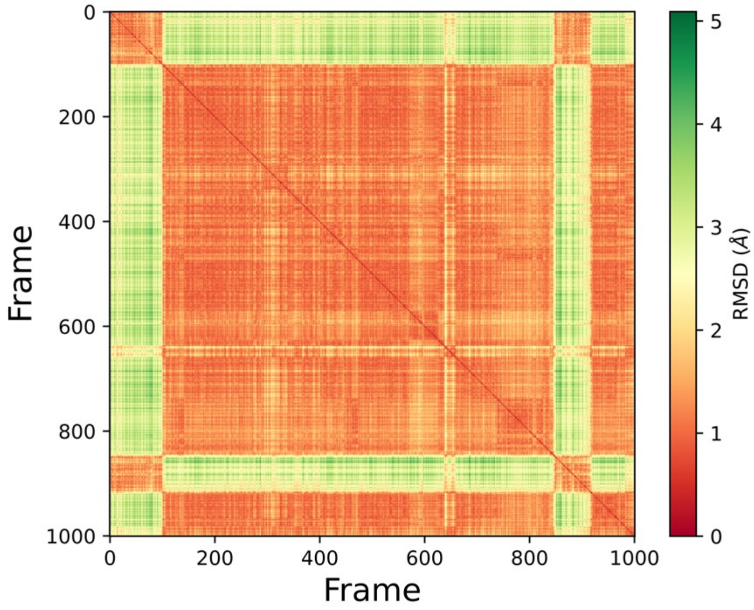

Figure S6. Pairwise RMSD for ZA-loop (76-106 residues) backbone atoms of the simulation in Figure 6A of the main manuscript.

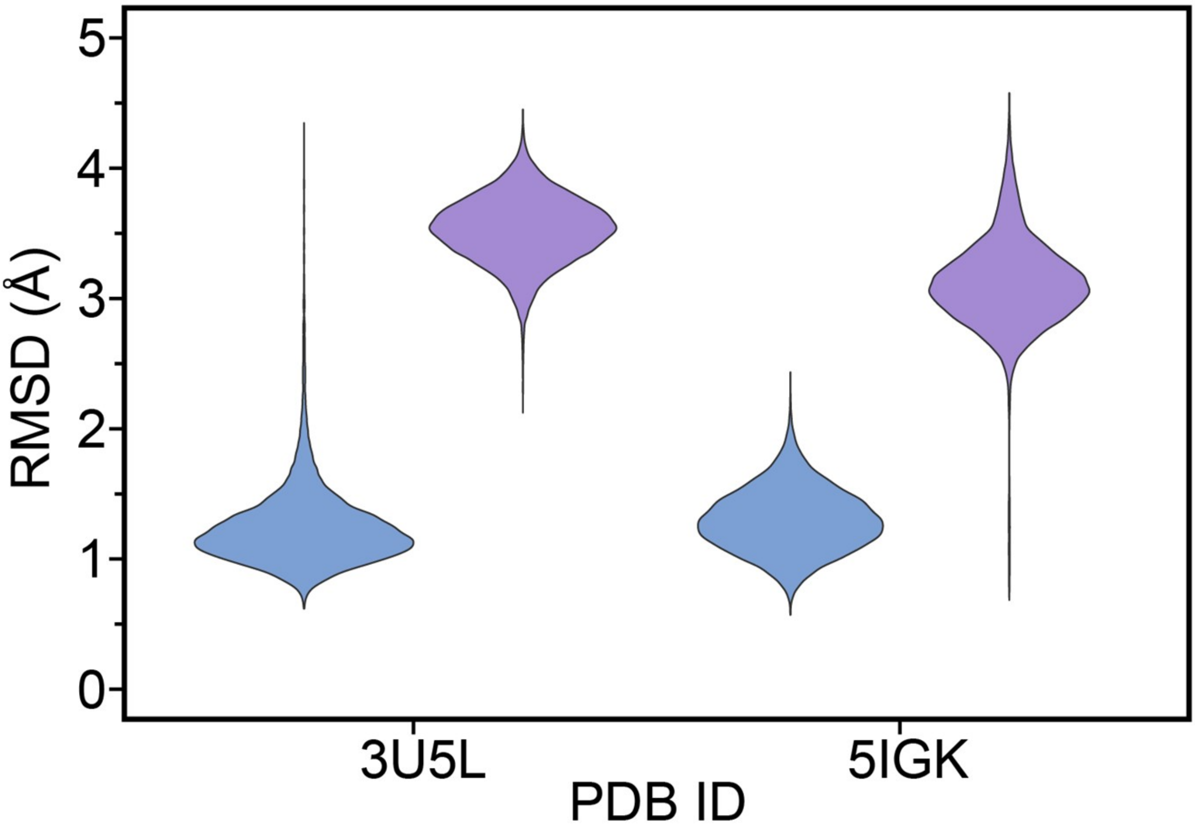

Figure S7. RMSD violin plots for ZA-loop (76-106 residues) backbone atoms of 3U5L and 5IGK for simulations having ZA1 (blue) and ZA2 (purple) in the starting structure.

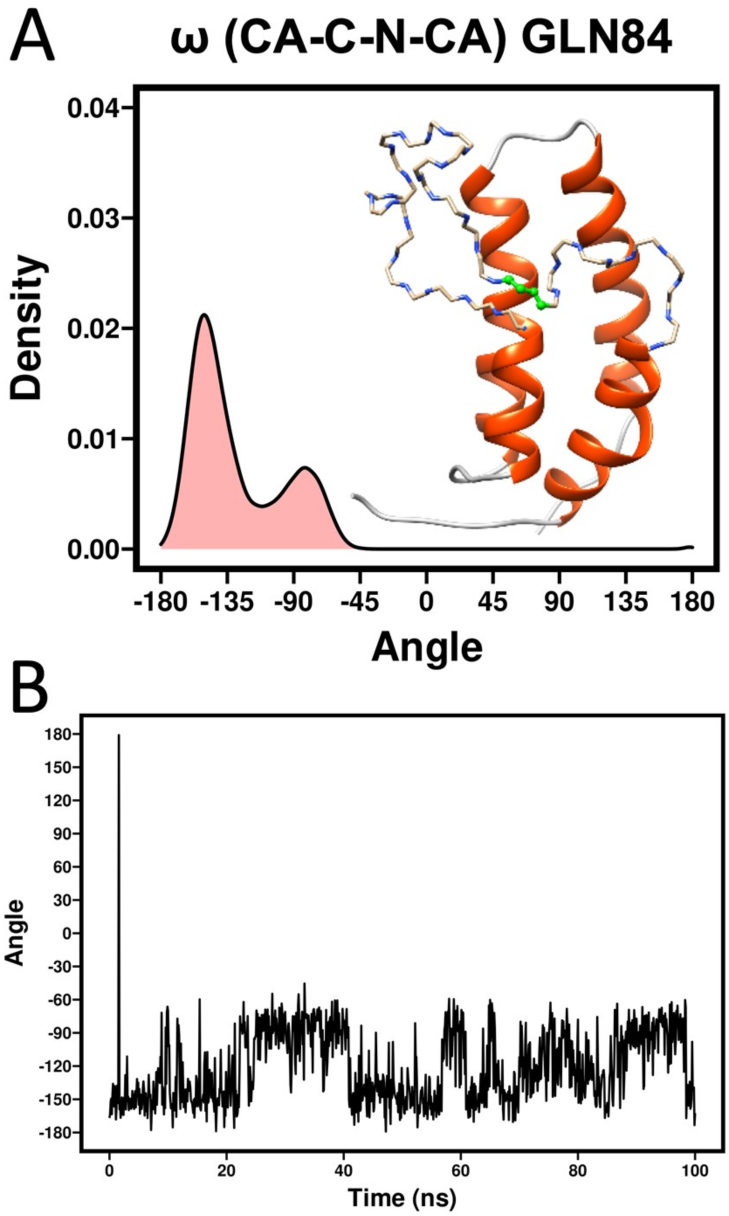

Figure S8. A) The ω (CA-C-N-CA) angle distribution of Gln84 (in the ZA loop) across 100 apo-receptor simulations.
Atoms are highlighted in green ball & stick in the inset cartoon. B) The angle change of the ω (CA-C-N-CA) Gln84 from the simulation in Figure 6A.

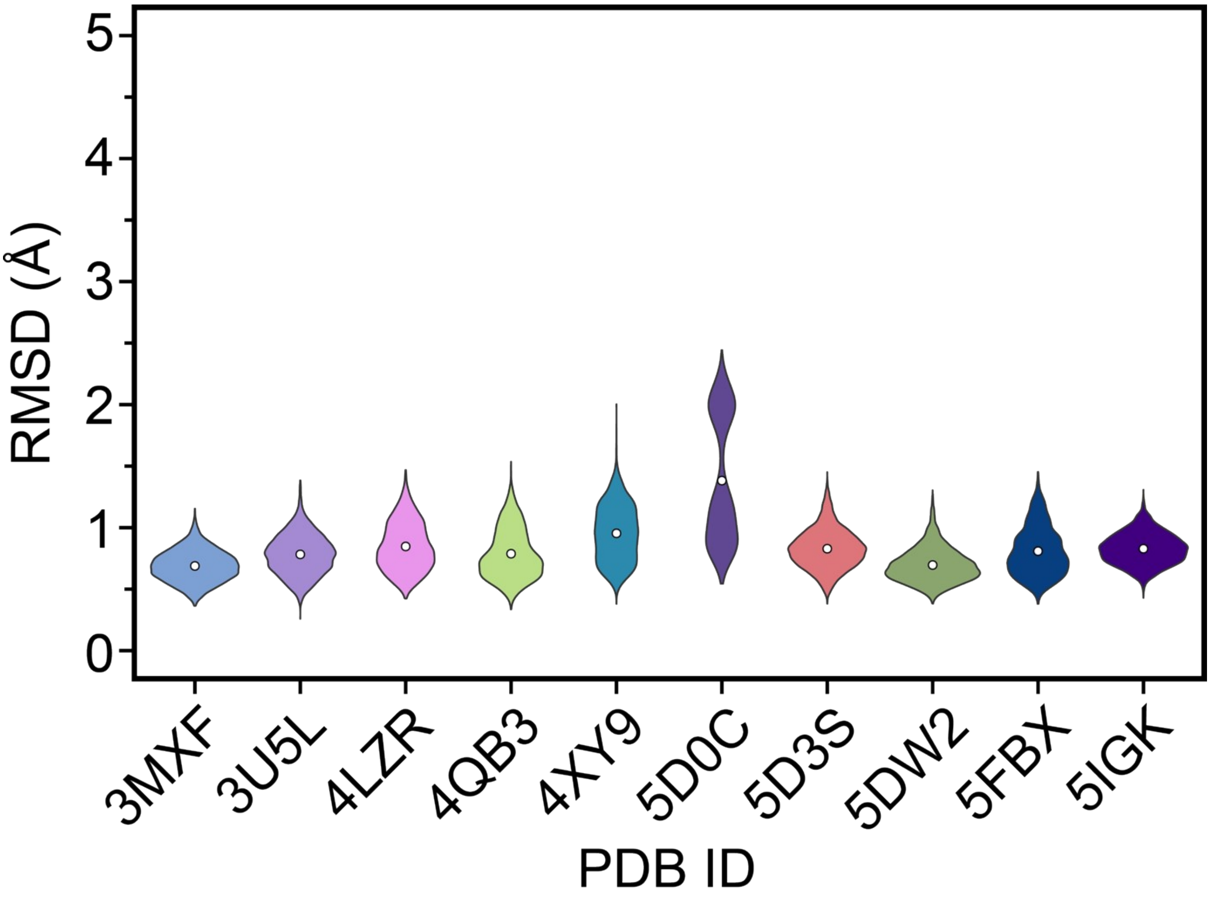

Figure S9. RMSD violin plots for the ZA-loop (76-106 residues) backbone atoms in crystal lattice simulations.

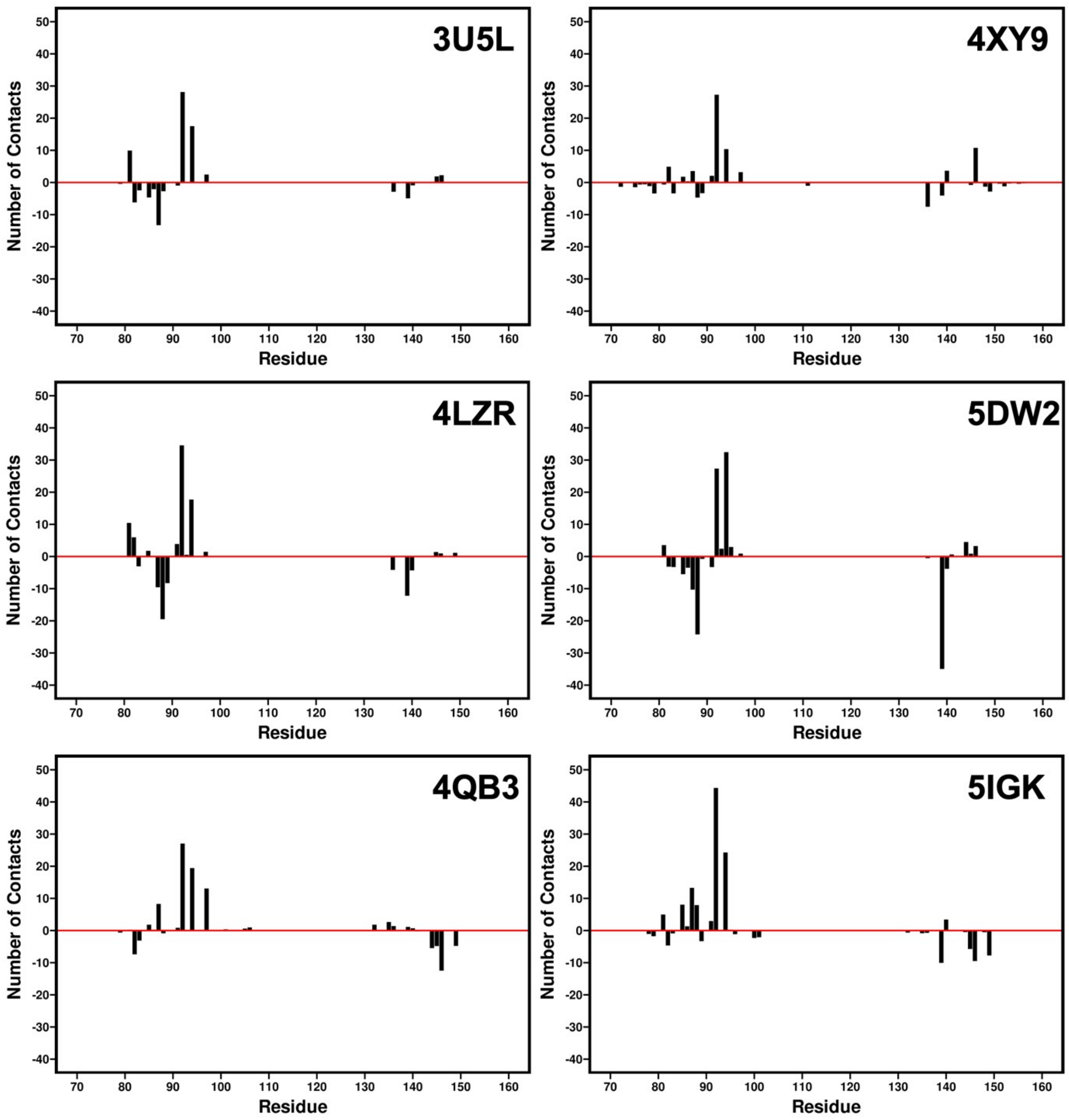

Figure S10. Difference (ZA1 – ZA2) of the average number of contacts between conformations.
Note that 5DW2 for which the ZA2 loop conformation gives the best agreement with experiment exhibits substantial contact preferences for Asp88 and Tyr139.

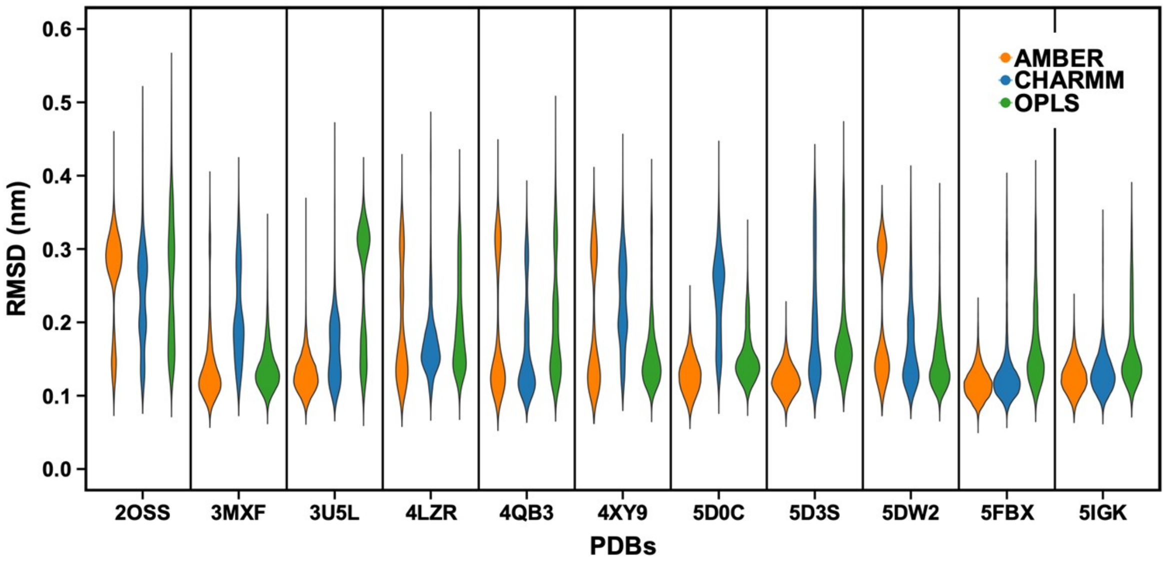

Figure S11. RMSD violin plots for ZA-loop (76-106 residues -see **Fig. 1**.) backbone atoms of 2OSS apo-receptor and complex simulations after fitting whole protein backbone.

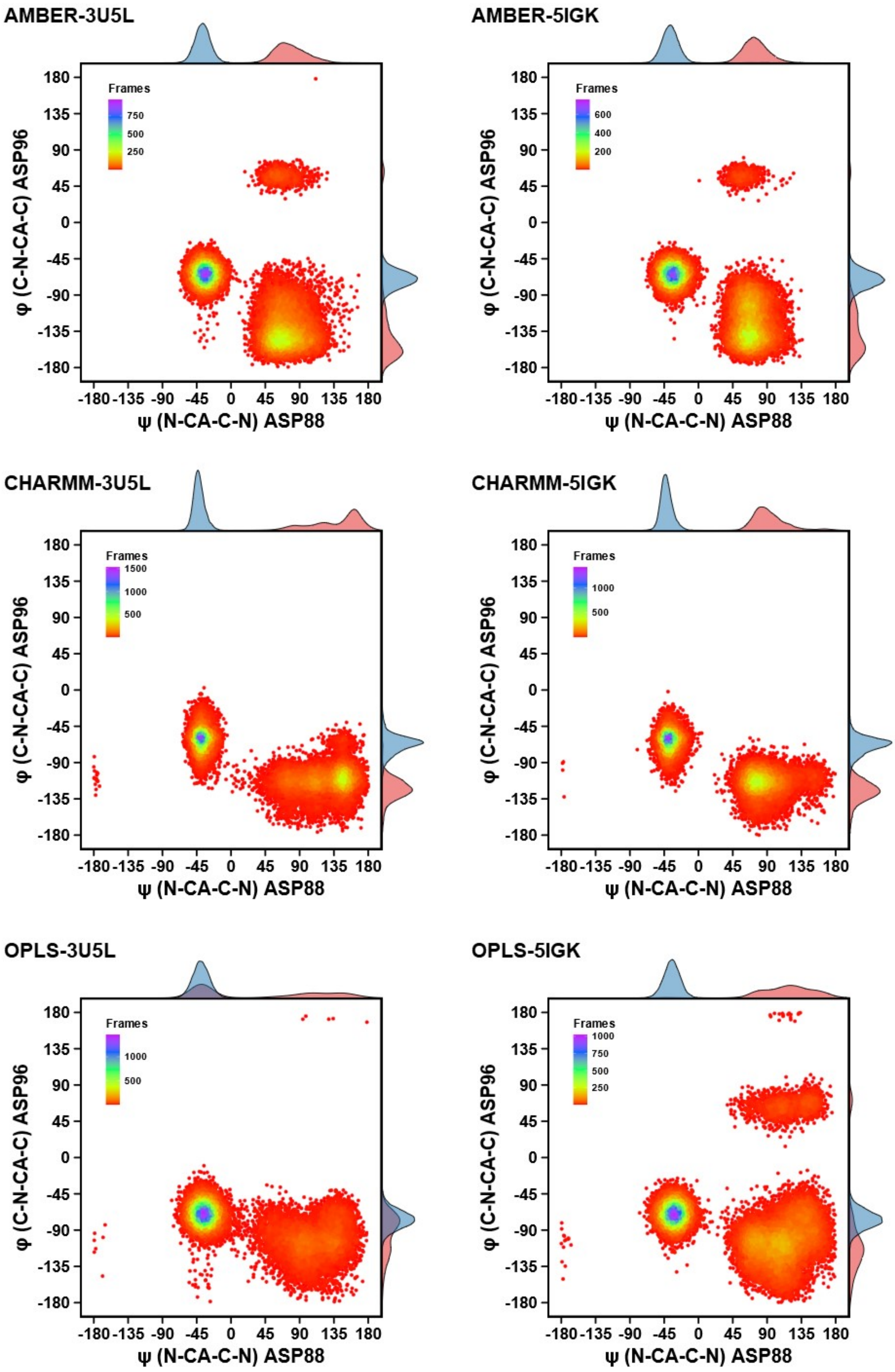

Figure S12. The overall sampling distribution of the simulations by dihedral angle distributions. Individual points from 40 simulations (ZA1+ZA2) are colored by the number of neighboring points. Marginal density plots on the top and right of the base plot show the distribution of dihedral angles in ZA1 (pink) and ZA2 (blue) simulations.

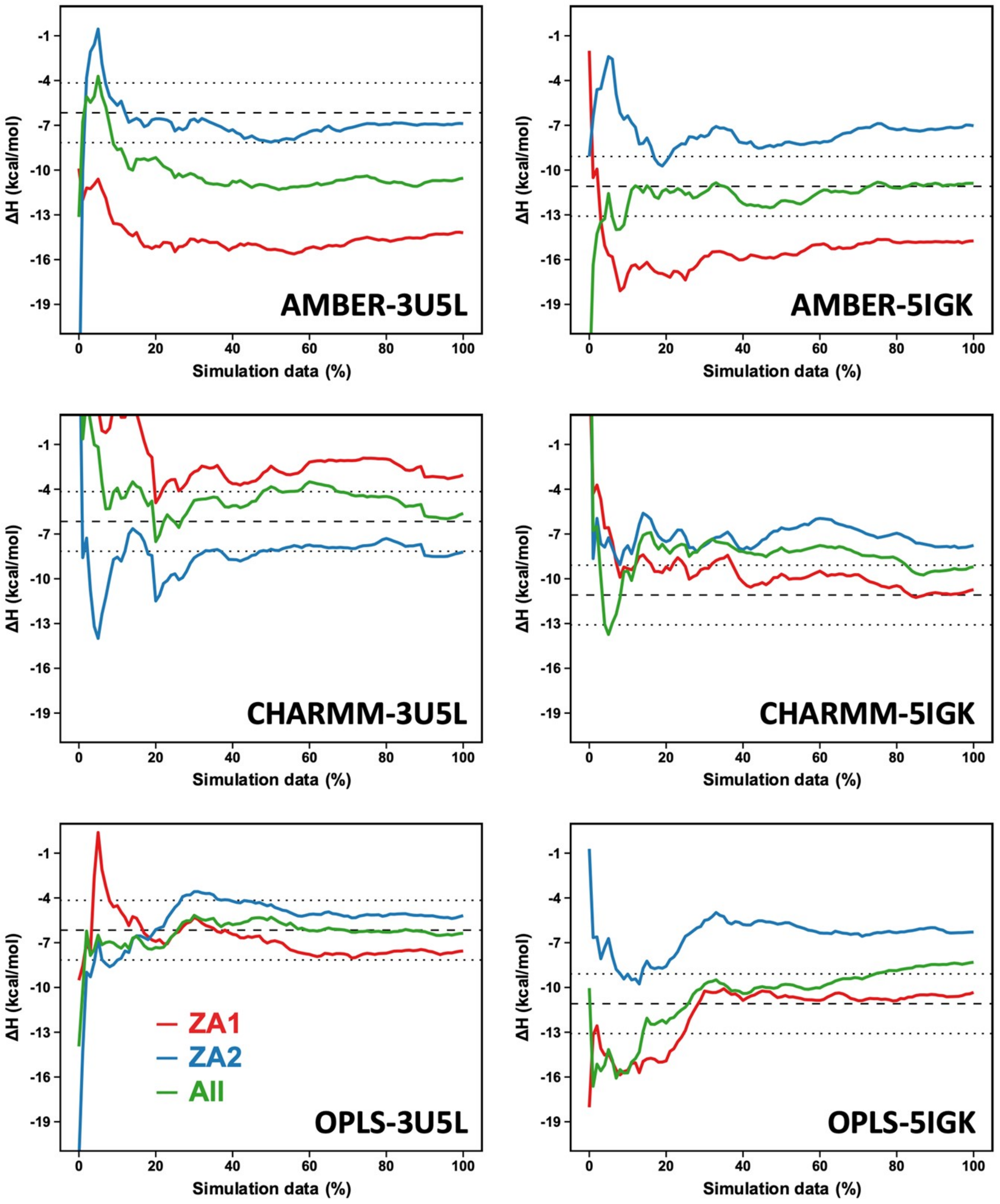

Figure S13. Convergence pattern of the calculated ΔH.
Red lines show convergence for the simulations with ZA1 (crystal-like conformation), blue lines are for the ZA2, and green lines show the convergence for all simulations consisting of ZA1 and ZA2 simulations. The dashed-line is the experimental ΔH and the dotted-lines indicate the 2 kcal/mol error limit.

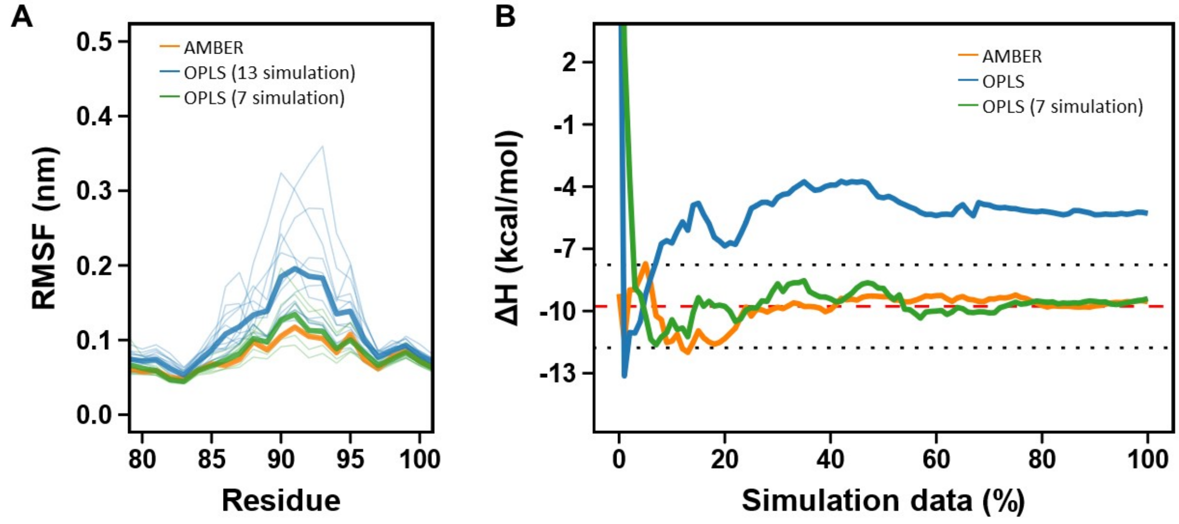

Figure S14: OPLS introduced greater flexibility to the ZA loop of 5D3S.
(A) RMSF for backbone atoms of ZA loop region. Bold lines show the average RMSF. (B) Convergence pattern of the calculated ΔH for 5D3S. The red dashed-line is the experimental ΔH and the dotted-lines indicate the 2 kcal/mol error limit.

REFERENCES

1. P. Filippakopoulos, J. Qi, S. Picaud, Y. Shen, W. B. Smith, O. Fedorov, E. M. Morse, T. Keates, T. T. Hickman, I. Felletar, M. Philpott, S. Munro, M. R. McKeown, Y. Wang, A. L. Christie, N. West, M. J. Cameron, B. Schwartz, T. D. Heightman, N. La Thangue, C. A. French, O. Wiest, A. L. Kung, S. Knapp and J. E. Bradner, *Nature*, 2010, **468**, 1067-1073.
2. M. G. J. Baud, E. Lin-Shiao, T. Cardote, C. Tallant, A. Pschibul, K. H. Chan, M. Zengerle, J. R. Garcia, T. T. L. Kwan, F. M. Ferguson and A. Ciulli, *Science*, 2014, **346**, 638-641.
3. P. Filippakopoulos, S. Picaud, O. Fedorov, M. Keller, M. Wrobel, O. Morgenstern, F. Bracher and S. Knapp, *Bioorganic & Medicinal Chemistry*, 2012, **20**, 1878-1886.
4. S. Picaud, D. Da Costa, A. Thanasopoulou, P. Filippakopoulos, P. V. Fish, M. Philpott, O. Fedorov, P. Brennan, M. E. Bunnage, D. R. Owen, J. E. Bradner, P. Taniere, B. O'Sullivan, S. Müller, J. Schwaller, T. Stankovic and S. Knapp, *Cancer Research*, 2013, **73**, 3336-3346.
5. S. Picaud, C. Wells, I. Felletar, D. Brotherton, S. Martin, P. Savitsky, B. Diez-Dacal, M. Philpott, C. Bountra, H. Lingard, O. Fedorov, S. Muller, P. E. Brennan, S. Knapp and P. Filippakopoulos, *Proceedings of the National Academy of Sciences*, 2013, **110**, 19754-19759.
6. K. G. McLure, E. M. Gesner, L. Tsujikawa, O. A. Kharenko, S. Attwell, E. Campeau, S. Wasiak, A. Stein, A. White, E. Fontano, R. K. Suto, N. C. W. Wong, G. S. Wagner, H. C. Hansen and P. R. Young, *PLoS ONE*, 2013, **8**, e83190.
7. X. Lucas, D. Wohlwend, M. Hügle, K. Schmidtkunz, S. Gerhardt, R. Schüle, M. Jung, O. Einsle and S. Günther, *Angewandte Chemie International Edition*, 2013, **52**, 14055-14059.
8. P. Ciceri, S. Müller, A. O'Mahony, O. Fedorov, P. Filippakopoulos, J. P. Hunt, E. A. Lasater, G. Pallares, S. Picaud, C. Wells, S. Martin, L. M. Wodicka, N. P. Shah, D. K. Treiber and S. Knapp, *Nature Chemical Biology*, 2014, **10**, 305-312.
9. M. Gacias, G. Gerona-Navarro, Alexander, G. Zhang, L. Zeng, J. Kaur, G. Moy, E. Rusinova, Y. Rodriguez, B. Matikainen, A. Vincek, J. Joshua, P. Casaccia and M.-M. Zhou, *Chemistry & Biology*, 2014, **21**, 841-854.
10. S. Picaud, M. Strocchia, S. Terracciano, G. Lauro, J. Mendez, D. L. Daniels, R. Riccio, G. Bifulco, I. Bruno and P. Filippakopoulos, *Journal of Medicinal Chemistry*, 2015, **58**, 2718-2736.
11. A. Hammitzsch, C. Tallant, O. Fedorov, A. O'Mahony, P. E. Brennan, D. A. Hay, F. O. Martinez, M. H. Al-Mossawi, J. De Wit, M. Vecellio, C. Wells, P. Wordsworth, S. Müller, S. Knapp and P. Bowness, *Proceedings of the National Academy of Sciences*, 2015, **112**, 10768-10773.
12. X. Xue, Y. Zhang, Z. Liu, M. Song, Y. Xing, Q. Xiang, Z. Wang, Z. Tu, Y. Zhou and K. Ding, *Journal of medicinal chemistry*, 2016, **59**, 1565-1579.
13. M. Hügle, X. Lucas, G. Weitzel, D. Ostrovskyi, B. Breit, S. Gerhardt, O. Einsle, S. Günther and D. Wohlwend, *Journal of medicinal chemistry*, 2016, **59**, 1518-1530.
14. O. A. Kharenko, E. M. Gesner, R. G. Patel, K. Norek, A. White, E. Fontano, R. K. Suto, P. R. Young, K. G. McLure and H. C. Hansen, *Biochemical and Biophysical Research Communications*, 2016, **477**, 62-67.
15. B. Raux, Y. Voitovich, C. Derviaux, A. Lugari, E. Rebuffet, S. Milhas, S. Priet, T. Roux, E. Trinquet and J.-C. Guillemot, *Journal of medicinal chemistry*, 2016, **59**, 1634-1641.
16. R. C. Montenegro, P. G. K. Clark, A. Howarth, X. Wan, A. Ceroni, P. Siejka, G. A. Nunez-Alonso, O. Monteiro, C. Rogers, V. Gamble, R. Burbano, P. E. Brennan, C. Tallant, D. Ebner, O. Fedorov, E. O'Neill, S. Knapp, D. Dixon and S. Müller, *Oncotarget*, 2016, **7**, 43997-44012.
17. S. Picaud, K. Leonards, J.-P. Lambert, O. Dovey, C. Wells, O. Fedorov, O. Monteiro, T. Fujisawa, C.-Y. Wang, H. Lingard, C. Tallant, N. Nikbin, L. Guetzoyan, R. Ingham, S. V. Ley, P. Brennan, S. Muller, A. Samsonova, A.-C. Gingras, J. Schwaller, G. Vassiliou, S. Knapp and P. Filippakopoulos, *Science Advances*, 2016, **2**, e1600760.

18. R. Galvelis, S. Doerr, J. M. Damas, M. J. Harvey and G. De Fabritiis, *J. Chem. Inf. Model.*, 2019, **59**, 3485-3493.
